# Distinct chemotactic behavior in the original *Escherichia coli* K-12 depending on forward-and-backward swimming, not on run-tumble movements

**DOI:** 10.1038/s41598-020-72429-1

**Published:** 2020-09-28

**Authors:** Yoshiaki Kinosita, Tsubasa Ishida, Myu Yoshida, Rie Ito, Yusuke V. Morimoto, Kazuki Goto, Richard M. Berry, Takayuki Nishizaka, Yoshiyuki Sowa

**Affiliations:** 1 Department of Physics, Gakushuin University, 1-5-1 Mejiro, Toshima-ku, Tokyo, 171-8588 Japan; 2 Department of Physics, University of Oxford, Park load, Oxford, OX1 3PU UK; 3 Department of Frontier Bioscience and Research Center for Micro-Nano Technology, Hosei University, Tokyo, 184-8584 Japan; 4 Department of Physics and Information Technology, Faculty of Computer Science and Systems Engineering, Kyushu Institute of Technology, Iizuka, Fukuoka Japan; 5 Present Address: Molecular Physiology Laboratory, RIKEN, Wako, Japan

**Keywords:** Microbiology, Bacteria, Cellular microbiology, Biophysics, Motility

## Abstract

Most motile bacteria are propelled by rigid, helical, flagellar filaments and display distinct swimming patterns to explore their favorable environments. *Escherichia coli* cells have a reversible rotary motor at the base of each filament. They exhibit a run-tumble swimming pattern, driven by switching of the rotational direction, which causes polymorphic flagellar transformation. Here we report a novel swimming mode in *E. coli* ATCC10798, which is one of the original K-12 clones. High-speed tracking of single ATCC10798 cells showed forward and backward swimming with an average turning angle of 150°. The flagellar helicity remained right-handed with a 1.3 μm pitch and 0.14 μm helix radius, which is consistent with the feature of a curly type, regardless of motor switching; the flagella of ATCC10798 did not show polymorphic transformation. The torque and rotational switching of the motor was almost identical to the *E. coli* W3110 strain, which is a derivative of K-12 and a wild-type for chemotaxis. The single point mutation of N87K in FliC, one of the filament subunits, is critical to the change in flagellar morphology and swimming pattern, and lack of flagellar polymorphism. *E. coli* cells expressing FliC(N87K) sensed ascending a chemotactic gradient in liquid but did not spread on a semi-solid surface. Based on these results, we concluded that a flagellar polymorphism is essential for spreading in structured environments.

## Introduction

The flagellar motor is the most extensively investigated motility system in bacteria^1–3^. The motor complex is composed of approximately 30 different proteins and is attached to the helical flagellar filament via a hook structure. Most flagellar motors rotate in both directions, and the rotating filament works as a screw to generate thrust against the surrounding medium^4,5^. Flagellated bacteria exhibit distinct chemotactic behaviors to move toward favorable environments. An *Escherichia coli* cell has 5–10 left-handed flagellar filaments protruding from its cell body; the rotation of a bundle of flagella in the counterclockwise (CCW) direction (when viewed from filament to motor) propels a cell forward^4,6^. The cell undergoes reorientation (tumbling) upon the switching of flagellar rotation from CCW to clockwise (CW), leading to a change in filament shape from left- to right-handed^7^. In the case of *Vibrio alginolyticus*, a single left-handed polar flagellum propels the cells forward and backward by CCW and CW rotation, respectively^8,9^. Additionally, *V. alginolyticus* cells change their swimming direction by ~ 90° due to a buckling instability of their straight hook (flick). Recently, a novel type of flagellar-wrapping motion in which the right-handed flagellum wraps around the cell body and propels the cell forward by its CW rotation has been discovered. This motility mode was found in *Magnetospirillum magneticus* AMB-1^10^, *Shewanella putrefaciens*^11^, *Pseudomonas putida*^12^*, Allivibrio fischeri*^13^*, Burkholderia insecticola*^13^, and *Helicobacter suis*^14^.

*E. coli* is an ideal model organism due to its rapid growth in pure nutrient media and the availability of many genetic tools to study it. Theodor Escherich, the German pediatrician, isolated *Bacterium coli* from the feces of healthy individuals in 1885; it was renamed *Bacillus coli* and eventually *E. coli* in 1919^15^. The original *E. coli* strain has been stored in the United Kingdom National Collection of Type Cultures as NCTC86; it shares a common genetic backbone with non-pathogenic *E. coli* strains*,* such as K-12, B, and HS^15,16^. In 1922, *E. coli* K-12 was isolated from the stool of a convalescent diphtheria patient^17^. Since then, many hundreds of K-12 derivatives have been isolated for motility studies^18,19^; in addition, some K-12 derivatives have lost resistance to bacteriophage λ and sexual fertility (F^+^), due to the effect of UV irradiation and acridine orange, during this period^17^. Initial studies isolated motile strains, such as W2637 and MG1655, using a semi-solid agar plate in which motile strains spread, but non-motile strains did not^20^. A recent study proposed navigated range expansion in *E. coli* cells as a dispersal mode in a structured environment (semi-solid agar plates). It has also been suggested as a population fitness mechanism that recognizes nutrients and chemical gradients that serve as local guides and that allows a rapid expansion into unoccupied territories (outer edge)^21^. However, the reason why the original K-12 strain exhibits no motility remains unclear; we infer that these non-motile cells have uncharacterized and exciting features to contribute to the field of bacterial flagellar studies.

Accordingly, we investigated the swimming motility of the original K-12 strain, ATCC10798. We could not observe any spreading on a semi-solid agar plate, but the cells exhibited swimming motility in liquid medium, with a forward and backward movement similar to that of *V. alginolyticus*. A single amino acid substitution seems to have prevented flagellar polymorphism and the consequent change of chemotactic behavior from run-tumble to forward–backward movements. We found that ATCC10798 cells could not swim in structured environments, although they could swim toward the attractant in liquids. Moreover, *E. coli* cells capable of a run and tumble strategy freely moved with 180° reversals to escape, if their route was blocked. From these results, we argue the importance of flagellar polymorphism for migration in structured environments.

## Results

### Differences in swimming pattern and flagellar structure between *E. coli* ATCC10798 and W3110

**Figure 1:**
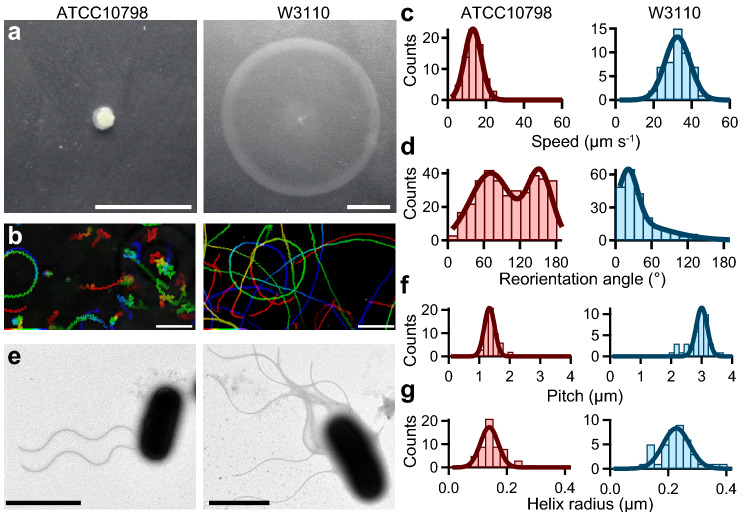
Characterization of swimming motility and structural parameters of ATCC10798 and W3110. (**a**) Motility of *E. coli* ATCC10798 and W3110 cells on 0.25% (wt/vol) soft-agar plates incubated at 30 °C for 7 h. Scale bar, 1 cm. (**b**) Sequential phase-contrast images taken at 50 ms intervals for 10 s were integrated using an intermittent color code: “red → yellow → green → cyan → blue.” Scale bar, 20 μm. (**c**) Histograms of the swimming speed of ATCC10798 (left) and W3110 (right). Solid lines represent the Gaussian fitting; the peaks (± SDs) are at 13.2 ± 4.4 μm s^−1^ in ATCC10798 (n = 70) and at 32.5 ± 6.6 μm s^−1^ in W3110 (n = 50). (**d**) Histogram of reorientation angles during swimming. The peaks (± SDs) are at 70 ± 31 degrees and 151 ± 23 degrees in ATCC10798 (n = 354) and at 34 ± 13 degrees in W3110 (n = 119). (**e**) Electron micrographs of *E. coli* ATCC10798 (left) and W3110 (right) cells. Scale bars, 2 μm. (**f**) Histograms of the flagellar pitch. Solid lines represent the Gaussian fitting, the peaks (± SDs) are at 1.3 ± 0.2 μm for ATCC10798 (n = 59) and at 3.0 ± 0.2 μm for W3110 (n = 41). (**g**) Histograms of the helix radius. The peaks (± SDs) are at 0.14 ± 0.03 μm in ATCC10798 (n = 59) and at 0.23 ± 0.05 μm in W3110 (n = 42).

We found that ATCC10798 cells did not spread on semi-solid agar plates, whereas W3110 cells were able to do so (Fig. 1a). Previous studies reported that cells defective in chemotaxis or motility, or lacking active flagella did not spread in this kind of medium (Supplementary Fig. 1)^22^; therefore, we hypothesized that ATCC10798 cells might be unable to exhibit either a swimming or a switching behavior. To address this, we performed microscopic measurements using a phase-contrast microscope (Supplementary Video 1). Unexpectedly, ATCC10798 cells showed swimming motility with reorientations, which is a different motility pattern from that of W3110 cells (Fig. 1b).

To better understand the different motility modes between strains, we quantified the swimming speed and switching pattern of individual cells. The average swimming speed (± standard deviation; SD) was 13.2 ± 4.4 μm s^−1^ in ATCC10798 cells and 32.5 ± 6.6 μm s^−1^ in W3110 cells (Fig. 1c). To characterize the switching behavior in detail, we calculated angle changes over time, between *θ*(*t*) and *θ*(*t* + Δ*t*), using a previously reported algorithm (see “Materials and methods” section)^23^. The frequency distribution of turning angles in ATCC10798 had a bimodal shape, with peaks at 70° and 150°, indicating that cells reversed their swimming direction (Fig. 1d left), as previously seen in a curly mutant of *Salmonella enterica serovar* Typhimurium^24^. The angle that was most frequently seen in W3110 cells when changing direction was close to 35°, similar to the previously reported angle (Fig. 1d right)^23^.

Next, we analyzed flagellar morphology using TEM. In ATCC10798, the average number of filaments was two, with an average length (± SD) of 4.7 ± 1.1 μm (Fig. 1e left, n = 62). The average flagellar pitch and helix radius were 1.3 ± 0.2 μm (Fig. 1f left) and 0.14 ± 0.03 μm (Fig. 1g left), respectively, which corresponded to a curly flagellar filament^25^. In W3110 cells, approximately six flagellar filaments were formed around the cell body (peritrichous flagella, Fig. 1e right). Their average length (± SD) was 7.3 ± 1.9 μm (n = 48), whereas their average pitch and helix radius were 3.0 ± 0.2 μm (Fig. 1f, right) and 0.23 ± 0.05 μm (Fig. 1g, right), respectively. These helical parameters indicated that these flagellar filaments belonged to the normal type^25^. We speculated that the differences in flagellar number between the two strains could be due to pipetting during sample preparation, given that cells with curly filaments tend to adhere to one another^26^ and that broken or detached curly filaments could be easily seen under TEM. Other structural parameters are summarized in Supplementary Table 1.

### Forward–backward movement in ATCC10798 swimming

To elucidate the basis for the difference in the swimming mode between ATCC10798 and W3110, we labeled the flagellar filaments with a fluorescent dye, Cy3, taking advantage of the biotin-avidin interaction (see “Materials and methods”)^27,28^. First, we observed flagellar dynamics in a large field (230 × 144 μm). ATCC10798 cells with few flagellar filaments frequently exhibited forward and backward swimming, like those in *V. alginolyticus*^8,9^, whereas cells with many filaments showed wobbling motion, apparently due to deficient bundle formation (Supplementary Video 2). Most W3110 cells exhibited directed linear motion (run) with abrupt directional changes (tumble)^6,23^.

**Figure 2:**
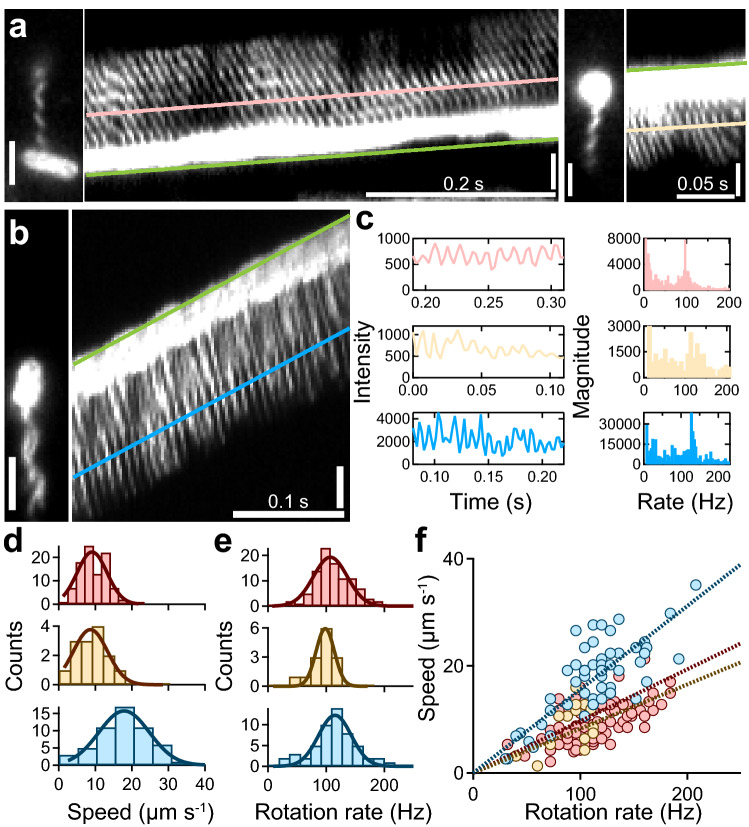
Visualization of forward and backward movements in ATCC10798. (**a**) Micrographs and kymographs of ATCC10798 cells during backward (left) and forward swimming (right). The green line drawn at the tip of the cell enabled quantification of the swimming speed of the cells. The pink and beige lines were drawn on the signal of flagella, where the slopes were the same as those of the green lines. Scale bar, 2 μm. (**b**) Typical example of a swimming run of W3110 cells. The green line drawn at the tip of the cell enabled quantification of the swimming speed of the cells. The blue line was drawn on the signal of flagella, where the slope was the same as that of the green line. Scale bar, 2 μm. (**c**) Left: intensity changes along flagellar filaments, colors correspond to those in (**a**) and (**b**). Right: frequency analysis was done by a Fourier transform. The peaks for backward and forward swimming of ATCC10798, and for the run of W3110 were at 94, 108, and 124 Hz, respectively. (**d**) Speed histograms of backward (top) and forward (middle) swimming of ATCC10798 cells, and of the swimming run of W3110 cells (bottom). The solid lines represent the Gaussian fitting, where the peaks (± SDs) are at 9.1 ± 4.1 μm s^−1^ for backward swimming (n = 96), at 8.7 ± 4.7 μm s^−1^ for forward swimming (n = 14), and at 17.9 ± 6.9 μm s^−1^ for the W3110 cell run (n = 54). (**e**) Flagellar rotation rate histograms show that the peaks (± SDs) are at 107.2 ± 29.1 Hz during backward swimming (top) and at 98.4 ± 15.7 Hz during forward swimming (middle) for ATCC10798 cells, and at 115.1 ± 28.1 Hz for W3110 cells (bottom). (**f**) Relationship between swimming speed and rotation rate. Colors correspond to those in (**d**) and (**e**). Dashed lines represent linear fitting with slopes of 0.083 μm per revolution during backward swimming and 0.097 μm per revolution during forward swimming for ATCC10798 cells, and 0.156 μm per revolution during swimming run for W3110 cells.

We next recorded both the swimming and flagellar rotation simultaneously at a high S/N ratio and a speed of 400 frames s^−1^ (Supplementary Video 3)^27,28^. Using kymograph analysis (see Materials and methods”), the swimming speeds and rotational rates were quantified from the slope of the resulting curve and the changes in intensity, respectively (Fig. 2a–c). In ATCC10798, the swimming speed and the rotation rate were estimated to be 9.1 ± 4.1 μm s^−1^ and 107.2 ± 29.1 Hz during backward swimming (Fig. 2a, left; Fig. 2d,e, top) and 8.7 ± 4.7 μm s^−1^ and 98.4 ± 15.7 Hz during forward swimming (Fig. 2a, right; Fig. 2d,e, middle), respectively. In W3110 (Fig. 2b), these parameters were 17.9 ± 6.9 μm s^−1^ and 115.1 ± 28.1 Hz (Fig. 2d,e, bottom), respectively. Despite having similar rotational rate values, flagellar rotation in W3110 cells was approximately twice as efficient than that of ATCC10798 cells: the swimming speed (*v*)/rotation rate (*f*) ratio was 0.156 μm/rotation in W3110, whereas it was 0.083 μm/rotation during backward swimming and 0.097 μm/rotation during forward swimming in ATCC10798 (Fig. 2f). These results suggest that a larger helix can produce a stronger thrust, as previously predicted by mathematical modeling^29^.

### Real-time imaging of structure and kinematics for flagellar filaments under TIRFM

We next determined flagellar structure and function, simultaneously, using TIRFM^13,27,28^. We found that a cell attached to the glass surface could rotate its flagellar filament freely, by treating coverslips with poly-L-lysine and bovine serum albumin (BSA). In ATCC10798, we could see wave propagation, away from the cell body, during CW rotation of right-handed flagellar filaments, and toward the cell body during CCW rotation (Supplementary Video 4). From this analysis, we conclude that forward and backward movements in ATCC10798 cells are driven by CW and CCW rotation, respectively, in right-handed flagellar filaments. ATCC10798 and W3110 flagellar morphology and rotation rates in both motility modes are summarized and compared in Supplementary Fig. 2.

Although ATCC10798 cells had only right-handed flagellar filaments, W3110 cells had both right- and left-handed flagellar filaments (Supplementary Video 5). Besides, W3110 cells mainly formed left-handed flagellar helices when the filaments freely rotated in the CCW direction. Motor switching caused gyration of the filament and transformation from a left-handed into a right-handed filament within 100 ms (Supplementary Fig. 3, left; and Supplementary Video 6). We also detected reversible transformation from right- to left-handed filaments (Supplementary Fig. 3, right; and Supplementary Video 7). Furthermore, we observed coiled-state flagellar filaments with a radius of 0.78 ± 0.02 μm in W3110 (Supplementary Fig. 4).

### Quantification of single motor behaviors by tethered-cell assay

**Figure 3:**
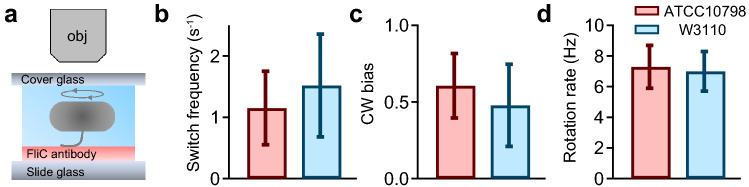
Quantification of switching behavior by tethered-cell assay. (**a**) Schematic representation of a tethered-cell assay. (**b**) Average (± SD) switching frequencies of ATCC10798 (1.15 ± 0.60 s^−1^; n = 99) and W3110 (1.52 ± 0.84 s^−1^; n = 53) cells. (**c**) Average (± SD) CW bias (Time_CW_/Time_Total_) for ATCC10798 (0.61 ± 0.21; n = 99) and W3110 (0.48 ± 0.27; n = 53) cells. (**d**) Average (± SD) rotation rates observed in ATCC10798 (7.3 ± 1.4 Hz; n = 99) and W3110 (7.0 ± 1.3 Hz; n = 53) cells (*P* = 0.1864 > 0.05 by *t*-test).

Previous studies claimed to identify torque-dependent flagellar transformation, based on direct measurements using a dark-field microscopy and molecular simulation^30,31^. However, we could not detect any flagellar transformation in ATCC10798 experiments, suggesting that a motor torque might be insufficient to cause flagellar transformation. To address this point, we quantified motor properties using a tethered-cell assay (Fig. 3a, see “Materials and methods”), recording rotation for 10 s in each measurement. Calculated switching frequencies and CW biases (CW time/total time) were 1.15 ± 0.60 s^−1^ and 0.61 ± 0.21 in ATCC10798 and 1.52 ± 0.84 s^−1^ and 0.48 ± 0.27 in W3110 cells, respectively (Fig. 3b,c). Whereas the rotation rates of ATCC10798 and W3110 were 7.3 ± 1.4 Hz and 7.0 ± 1.3 Hz, respectively (Fig. 3d). We could not detect any difference in the motor speed between the two strains (*P* = 0.1864 > 0.05 by *t*-test), suggesting that the defective flagellar polymorphism of ATCC10798 was not caused by its motor properties.

### Single-point mutation FliC (N87K) is essential for forward–backward movement

**Figure 4:**
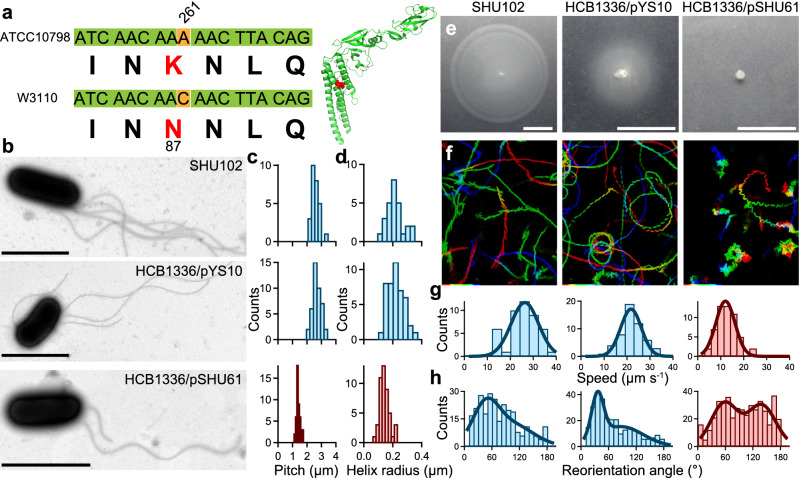
The FliC (N87K) substitution alters flagellar shape and swimming mode. (**a**) Left: *fliC* gene and protein sequence comparison. Right: Crystal structure of FliC (PDB: 1IO1). The red structure represents N87 residue. (**b**) Electron micrographs of SHU102 [*fliC* (N87K):: *fliC,*], HCB1336/pYS10, and HCB1336/pSHU61. Scale bars, 2 μm. (**c**) Histograms of the flagellar pitch observed in SHU102 (top), HCB1336/pYS10 (middle), and HCB1336/pSHU61 (bottom) cells with peaks (± SDs) at 2.5 ± 0.2 μm (n = 27), 2.7 ± 0.3 μm (n = 42), and 1.4 ± 0.1 μm (n = 46), respectively. (**d**) Histogram of the helix radius observed in SHU102 (top), HCB1336/pYS10 (middle), and HCB1336/pSHU61 (bottom) cells with peaks (± SDs) at 0.20 ± 0.04 μm (n = 27), 0.22 ± 0.06 μm (n = 44), and 0.14 ± 0.03 μm (n = 46), respectively. (**e**) Cell motility on 0.25% (wt/vol) soft-agar plates after 7 h at 30 ºC. Scale bar, 1 cm. (**f**) Swimming traces at 150 ms intervals for 15 s. The intermittent color code indicates the time course from red to blue. Area, 68.6 × 85.9 μm. (**g**) Swimming speed histograms of SHU102 (left), HCB1336/pYS10 (middle), and HCB1336/pSHU61 (right) cells with peaks (± SD) at 26.3 ± 6.0 μm s^−1^ (n = 45), 21.7 ± 4.5 μm s^−1^ (n = 53), and 12.0 ± 4.2 μm s^−1^ (n = 53), respectively. (**h**) Reorientation angle histograms with peaks (± SDs) at 46 ± 28 degrees in SHU102 cells (left, n = 269), at 36 ± 13 degrees in HCB1336/pYS10 cells (middle, n = 285), and at 58 ± 29 degrees and 139 ± 30 degrees in HCB1336/pSHU61 cells (right*,* n = 444).

The bistable protofilament model explains polymorphic flagellar transition. Basically, the flagellar filament is composed of 11 protofilaments, each of which assumes either a left or right conformation; different mixtures of these two types of protofilaments form filaments of up to12 different geometries/helical shapes^32–34^. Additionally, it is known that some point mutations can lead to the formation of these left and right type protofilaments^35–37^. Therefore, we compared the flagellin, FliC, sequences of ATCC10798 and W3110 and found that residue 87 in ATCC10798 was a lysine and not an asparagine as in W3110 (Fig. 4a). Note that we included the first methionine residue in the sequence of FliC although it is cleaved off post-translationally in *S. typhimurium*^38^. The effects of several amino acid substitutions on polymorphic flagellar transformation is well studied, but, to our knowledge, the effect of this FliC (N87K) substitution on flagellar formation has never been investigated.

To check whether this substitution was truly responsible for the transformation from a left-handed to a right-handed flagellar filament, we replaced the *fliC* gene of ATCC10798 with a wild type one, strain SHU102 [ATCC10798 (*fliC* (N87K):: *fliC*)] (see “Materials and methods”). We first examined the flagellar morphology of SHU102 using TEM (Fig. 4b, top). Then, we determined that the pitch and helical radius of SHU102 flagella were 2.5 ± 0.2 μm (Fig. 4c, top) and 0.20 ± 0.04 μm (Fig. 4d, top), respectively, which corresponded to those of the normal flagellar type, as observed in W3110 (Fig. 1f,g, right). We next investigated the swimming motility of SHU102 on semi-solid agar plates and found that it spread in a similar way than strain W3110, *i*.*e*., both strains formed migration halos with similar diameters (Fig. 4e). Additionally, SHU102 cells displayed a run and tumble pattern in its chemotactic behavior (Supplementary Videos 8–9 and Fig. 4f–h, left). Furthermore, we examined the effect of this substitution on the chemotactic response of the strain, using a capillary (tip) assay, and found that the FliC(N87K) substitution did not have any significant influence on it (Supplementary Video 10 and Supplementary Fig. 5).

We also examined the rotation rate and morphology of the flagellar filaments using TIRFM (Supplementary Fig. 6) and found that SHU102 cells had left-handed flagellar filaments. The flagellar helicity frequently underwent switching into a right-handed form, depending on motor switching, which had never been observed in ATCC10798 cells (Supplementary Video 11). These results suggest that FliC (N87K) caused the structure of filaments to be fixed in a right-handed helicity.

To confirm the effect of the FliC (N87K) substitution on motility and flagellar structure, we carried out a series of experiments on a Δ*fliC* strain, HCB1336, carrying either pYS10 (pBR322-*fliC*) or pSHU61 (pBR322-*fliC* (N87K)) (see “Materials and methods”). HCB1336/pYS10 formed normal flagellar filaments similar to those formed by W3110 and SHU102 cells (Fig. 4b–d, middle), while HCB1336/pSHU61 formed curly filaments like those in ATCC10798 cells (Fig. 4b–d, bottom). The swimming abilities, including spreading, swimming speed, and reorientation angle, of HCB1336/pYS10 and HCB1336/pSHU61 were similar to those of W3110 and ATCC10798 cells, respectively (Fig. 4e–h). We also visualized flagellar morphology and function using tethered-cell assays and TIRFM (Supplementary Figs. 7–8 and Supplementary Video 11). Resulting parameters are summarized in Supplementary Table 1.

### W3110 cells can escape from stuck on agarose surface through 180° reverse movements

**Figure 5:**
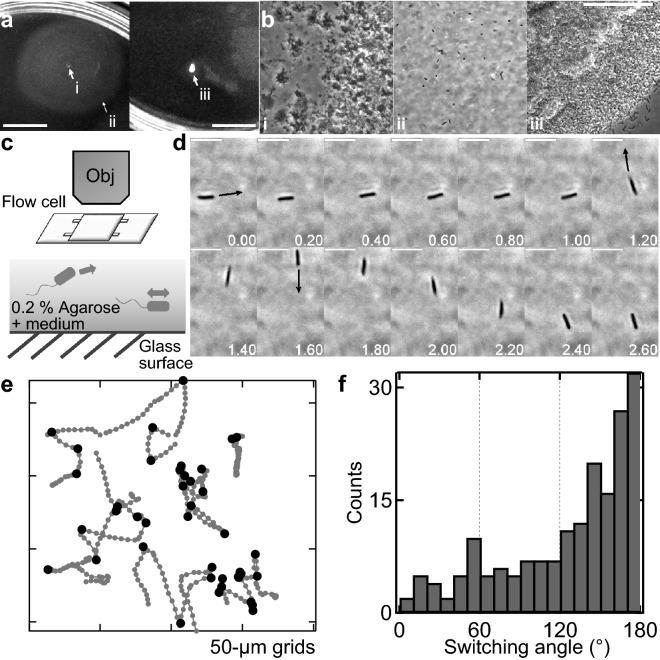
Swimming motility on 0.2% agarose. (**a**) Motility of W3110 (left) and ATCC10798 (right) cells on 0.2% (wt/vol) soft-agarose plates after incubation at 30 °C for 7 h. (**b**) Magnified images of the i, ii, and iii spots shown in (**a**). These experiments were conducted on the same plate. Scale bars, 1 cm (**a**) and 50 μm (**b**). (**c**) Top: Schematic representation of the observation of bacterial swimming motility in a 0.2% soft agarose pad. Bottom: Schematic representation of W3110 cells swimming in a medium containing 0.2% agarose. (**d**) Sequential images of W3110 cell migration in 0.2% agarose. Arrows indicate bacterial swimming direction after reversal, angle changes were approximately of 180 degrees each. Scale bar, 5 μm. (**e**) Typical examples of swimming trajectories with turning events. Black dots indicate the moments where reversals have taken place. Intervals, 20 ms. (**f**) Histogram of the switching angles observed in W3110 cells swimming in a 0.2% agarose medium (n = 183).

Although ATCC10798 cells showed chemotaxis in a liquid environment (Supplementary Fig. 5), they were not able to spread on semi-solid agar (Fig. 1). To determine the reason of this behavior, we checked swimming motility using agarose. As with the agar experiment, ATCC10798 cells could not spread on a 0.2% agarose plate, but W3110 cells could (Fig. 5a). Phase-contrast microscopy revealed that some W3110 cells were sparsely distributed along the migration halo (Fig. 5bi,ii), whereas ATCC10798 cells were densely packed in the center of the plate (Fig. 5biii). To check this difference in detail, we observed the swimming motility of fresh cells using a 0.2% semi-solid agarose pad (Fig. 5c, see “Materials and methods”). ATCC10798 cells were not able to swim once they stuck to the surface (Supplementary Video 12). In contrast, W3110 cells frequently stuck to the surface but escaped via 180° reversals, without reorientation of the cell body (Fig. 5d–f). Turner et al. also observed this phenomenon using a fluorescent microscope: the flagellar bundle transformed from a normal to a curly state and the curly filaments formed a bundle that pushed the cell forward, to the opposite side of the original direction of swimming^39^. Additionally, we found that W3110 cells frequently reversed their swimming direction in the presence of 15% (w/vol) Ficoll, as seen in constricted environments^40^; ATCC10798 cells were also able to swim with a forward and backward movement (Supplementary Video 13). Taken together, these results suggest that flagellar polymorphism is essential for migration in structured environments.

## Discussion

The FliC (N87K) substitution caused flagellar transformation from a left-handed, normal flagellar filament into a right-handed, curly filament (Fig. 4). Flagellin monomer consists of four connected domains, D0-D3 (Supplementary Fig. 9a). The highly conserved D0 and D1 domains face inward into the filament core, while D2 and D3 domains protrude outside, against the central core (Supplementary Fig. 9b)^41^. The role of D2-D3 is to stabilize flagellar filaments. D0-D1 are mainly responsible for L/R switching in protofilaments^26,41,42^. In flagellar filaments of *S. typhimurium* (and *E. coli*) amino acid substitutions in the D1 domain at A49, D108, D152, A415 (A417), A428 (A430), N434 (N436), and A450 (A452) cause flagellar transformation from a normal to a curly state^35–38^ (Supplementary Fig. 9a). These substitutions change the hydrogen-bonding network for the L/R transition along the 5-, 11-, and 16-start filament interfaces. We checked the interactions, highlighting hydrogen bonding interactions, between subunits using L- and R-type straight filaments of *S. typhimurium*^41,43^ as models (Supplementary Fig. 9c). In the L-type filament, the E84 and E122 residues form multiple hydrogen bonds with the N439 residue at the 5-start interface. In the R-type filament, hydrogen bonds are formed between E84 and T438 and between T130 and N439 at the 5-start interface. These residues are conserved among different bacterial species (Supplementary Fig. 9d). Although no specific interactions of the N87 residue were detected in L- or R-type filament subunits, the N87K mutation is likely to cause the formation of new hydrogen bonds with the T438 residue at the 5-start interface. This hydrogen bond might enhance R-type interactions and cause the adoption of the right-handed helical form, as previously shown^36^.

On a semi-solid agar plate, W3110 cells could spread, but ATCC10798 cells could not (Fig. 1). Spreading is generally associated with a chemotactic behavior driven by motor switching^22^. However, we infer that additional mechanisms are required for spreading on semi-solid agar plates taking into consideration that ATCC10798 cells exhibit both motor switching (Figs. 2, 3) and chemotactic behavior in liquid media (Supplementary Fig. 5). Interestingly, W3110 cells exhibited 180° reverse movements to escape from being stuck when in semi-solid agarose (Fig. 5), which is also observed in the peritrichous flagellated bacterium *Bacillus subtilis*^44^. Additionally, Turner et al. observed flagellar transformation-dependent 180° reversal movements using a fluorescent microscope (see fig. 5 in Ref.^39^) in structured environments^40,44^. Considering that the flagellar morphology was stable, irrespective of motor switching (Supplementary Video 4), we concluded that flagellar polymorphism was essential for spreading in structured environments. This idea enables us to easily interpret previous reports: specific point mutations in FliC, causing a lack of flagellar polymorphism, hinder the ability to swim on semi-solid agar plates, but still allow bacterial movement in liquid media^35,37^.

This mechanism could also be present in other types of flagellated bacteria. For example, flagellar polymorphic changes from a normal to a curly state have been seen in the single polar flagellated species *Pseudomonas* spp.^45,46^ as well as changes from a normal to a coiled state in the sub-polar flagellum in *Rhodobacter sphaeroides*^47^. A novel type of flagellar wrapping motion has recently been observed in polar-flagellated bacteria^10–14^. These bacteria reverse their direction of motion by transiting from a CCW rotation of left-handed normal filaments into a CW rotation of right-handed coiled filaments to escape from being trapped in structured environments. In *S. putrefaciens, flaB* is crucial, not only for flagellar polymorphism, but also for the transition from regular swimming to wrapping motion. However, cells with flagellar filaments consisting only of FlaA are deficient in both swimming motility in semi-solid agar and in flagellar polymorphism^48^. In common with *S. putrefaciens*, polar-flagellated bacteria possess multiple flagellins for flagellar polymorphism and migration in structured environments^49–51^. These data support that the ability of bacteria to swim in structured environments is driven by flagellar polymorphism. However, *Caulobacter crescentus* and *Vibrio* sp. spread on semi-solid agar plates without flagellar polymorphism^45,50,52^. In these bacteria, the hydrodynamic load causes the buckling of the straight hook upon a motor switching from CW to CCW rotation^8,9,53^. This buckling mechanism could be equivalent to flagellar polymorphism as a means to perturb cell motile patterns. In fact, a poly-hook mutant of non-chemotactic cells forms a pseudoring, driven by dynamic flagellar reorientation^54,55^. Our results complement recent, beautiful work on how microorganisms migrate in structured environments^21^ and will lead to a discussion of how *E. coli* cells have adapted for survival through the evolution of flagellar transformation.

## Materials and methods

This article was previously published as a preprint^56^.

### Bacterial strains

*E. coli* K-12 ATCC10798 and W3110 were the main strains used in this study. Other strains used are listed in Supplementary Table 2. Cells were grown at 37 °C on 1.5% (wt/vol) agar plates (010-08725; Wako) containing T-broth (1% (wt/vol) tryptone, 0.5% NaCl). Single colonies were isolated and resuspended in 10 mL of either T-broth or LB (1% (wt/vol) tryptone, 0.5% yeast extract, 0.5% NaCl) liquid medium^7^. Cells were grown to an optical density of 0.4–0.7 at 600 nm with shaking at 30 °C (Supplementary Fig. 10).

### Construction of *fliC* mutants

Plasmids and primers used in this study are listed in Supplementary Tables 2, 3. We purified genomic DNA from ATCC10798 and amplified the *fliC* gene by PCR. The *fliC* genes from ATCC10798 and W3110 only differed by one base corresponding to amino acid 87.

To determine the effect of the mutation at residue 87 on flagellar morphology, we performed two independent experiments: (i) the complementation of a Δ*fliC* strain with a plasmid encoding FliC (N87K); and (ii) the replacement of the chromosomal ATCC10798 *fliC* gene with an *E. coli* wild-type *fliC* gene. Plasmid pYS10 encodes a wild-type FliC; it was used to generate a plasmid encoding FliC (N87K) by the “QuikChange” site-directed mutagenesis method using 1217_fliC (N87K)-f(QC) and 1218_fliC(N87K)-r(QC).

Chromosomal substitution was achieved by using a λ Red recombination system, with plasmid pKD46 encoding the Red system^57^ and positive selection for the loss of tetracycline resistance^58^. The tetracycline-resistance gene *tetRA* was amplified by PCR using primers 0196_fliC-tetRA-F and 0197_fliC-tetRA-R. The *tetRA* cassette was replaced in the chromosomal *fliC* locus of the ATCC10798. After selection and isolation, SHU101 [*fliC* (N87K):: *tetRA*] was obtained and confirmed by colony PCR using 0219_fliC-(-175)-F, 0220_fliC-(+ 250)-R, 0210_tetRA-785-R, and 0211_tetRA-1090-F (Supplementary Fig. 1). *tetRA* in SHU101 was replaced by the wild-type *fliC* from the chemotactic wild-type strain RP437, which was amplified by PCR using primers of 1232_fliC-F and 0199_fliC-R. Tetracycline-sensitive clones were selected using tetracycline-sensitive plates and isolated as SHU102 [*fliC* (N87K):: *fliC*]. The construction was confirmed by sequence analysis using 0219_fliC- (-175)-F, 0220_fliC- (+ 250)-R, 0198_fliC-F, and 0199_fliC-R.

### Preparation of fluorescent-labeled cells

Cells were collected from 1 mL of culture by centrifugation at 6,000 *g* for 4 min at 25 °C, resuspended with buffer A (30 mM NaCl, 70 mM KCl, 2 mM EDTA), pH 7.8, containing biotin-NHS-ester, and incubated for 15 min at room temperature. After labeling, two rounds of centrifugation under previously mentioned conditions were done to remove excess biotin. Biotinylated cells were resuspended into buffer (30 mM NaCl, 70 mM KCl, 5 mM MgCl_2_), pH 7.0, containing 0.1 mg/mL Cy3-conjugated streptavidin and incubated for 3 min^27,28^. Excess dyes were removed by two more rounds of centrifugation and cells were resuspended into buffer B.

### Electron microscopy

Carbon-coated electron microscope grids were glow-discharged with a hydrophilic treatment device (PIB-10; Vacuum Device)^13,28^. Cells in buffer B were placed on the grid, incubated for 10 min at room temperature, and chemically fixed with 2% (vol/vol) glutaraldehyde in buffer B for 15 min. Cells were then washed three times with buffer B and subsequently treated by 2% (wt/vol) ammonium molybdate for staining. Samples were observed under a TEM (JEM-1400; JEOL) at 100 kV. Whole images were captured by a CCD camera at 8 bits.

### Motility assay on soft-agar plates

A single colony was inoculated on a 0.25% (wt/vol) T-broth soft-agar plate and incubated at 30 °C for 7 h. Cell motility was evaluated according to the colony’s diameter by Image J 1.45 s (https://rsb.info.nih.gov/ij/).

### Motility assay

All experiments were performed at room temperature. The flow chamber was composed of two coverslips (no. 1, 0.12–0.17 mm thickness) of different sizes (18 × 18 and 24 × 36 mm)^59,60^. The 24 × 36 mm cover glass was glow-discharged with a hydrophilic treatment device (PIB-10; Vacuum Device) to clean its surface. Double-sided tape pieces, ~ 30 mm long, were used as spacers between coverslips; they were fixed with ~ 5 mm intervals. The chamber’s final volume was ~ 7 μl, indicating that the double-sided tape was ~ 90 μm thick. For swimming assays, buffer C (30 mM NaCl, 70 mM KCl, 5 mM MgCl_2_, 5 mg/mL BSA was infused into the flow chamber, followed by 10 μL of the cell suspension medium.

For observation of stuck cells under total internal reflection fluorescence microscopy (TIRFM), a glass was coated with poly-L-lysine. Cells in buffer D (30 mM NaCl, 70 mM KCl, 5 mM MgCl_2_, 1.5 mg/mL BSA) were infused into the chamber, followed by 20 μL of buffer D to remove unbound cells.

A capillary assay was performed by the method of Nikata et al.^61^, using 10 μL tips as capillary tubes containing 5 μL of buffer B with 1% (wt/vol) agarose. Tips were inserted into a chamber for chemotactic response assays (Supplementary Fig. 5). Buffer C was infused into the chamber to prevent cells from adhering to the glass surface.

For tethered-cell assays, cell suspensions with an optical density of 0.6–0.8 at 600 nm were sheared by passing them back and forth 35 times through 1 mL syringes equipped with two 26 gauge needles connected by a piece of tubing. Cells were collected by centrifugation at  3,300 *g* for  3 min at 25 °C, resuspended in buffer E (10 mM KPi, 85 mM NaCl, 0.1 mM EDTA), washed twice, and resuspended in buffer E. Cells were then stuck on a glass surface via an anti-FliC antibody (1:300 dilution) (Fig. 3a); unbound cells were washed with buffer F (10 mM KPi, 67 mM NaCl, 0.1 mM EDTA, 10 mM lactate). Spinning cells were captured at 60 frames s^−1^ for 10 s through the 40 × objective, as previously described^5,62^. Rotational motions of cell bodies were analyzed using custom software-based upon LabVIEW (National Instruments). The CW bias was defined as $\frac{\mathrm{CW time }}{\mathrm{total time}}$ (Fig. 3c).

### Microscopy

For visualization of fluorescent-labeled cells, a green laser beam (λ = 532 nm; Compass-315 M-100, Coherent) was introduced into an inverted microscope (IX71, Olympus) equipped with a 100 × objective (Plan Apo TIRF, NA 1.49, Nikon Instruments), a dichroic mirror (custom-made, Chroma), an emission filter (NF01-532U, Semrock), an EMCCD camera (iXon + DU860, Andor), a CCD camera (HR1540; Digimo), a highly stable customized stage (Chukousha), and an optical table (RS-2000, Newport). Images were recorded at 2.5 ms intervals, using an EMCCD camera with a magnification of 130 × 130 nm at the single pixel on the camera plate.

### Live-cell imaging on agarose

We conducted two independent experiments to investigate swimming motility on 0.2% semi-solid agarose. First, Petri dishes with 0.2% agarose (20 mL) were inoculated with single colonies and incubated for 7 h at 30 °C (Fig. 5a,b). Then, 20 μL samples of 0.2% agarose were poured onto glass slides. When agarose had solidified, 10 μL of culture was added to each pad and covered with 22 × 22 mm coverslips using a double-sided tape with a ~ 20 mm interval and an approximate height of 75 μm (Fig. 5c). W3110 cells were used as positive swimming motility controls. Both experiments were visualized using an upright microscope (Eclipse Ci; Nikon) with a 40 × objective (EC Plan-Neofluar 40 with Ph and 0.75 N.A.; Nikon) and a CMOS camera (H1540; Digimo). Images were recorded at 20 fps for 15 s.

### Data analysis

To identify reorientation events within trajectories, we used three strategies, as previously reported^23^. First, phase-contrast images were captured at up to 200 frames s^−1^. The centroid position of cells determined swimming trajectories. Given the trajectory of cells, *r*(*t*) = [*x* (*t*), *y* (*t*)], the swimming velocity *v* (*t*) was defined as *v* (*t*) = $\frac{{\varvec{r}}\left({\varvec{t}}\boldsymbol{ }+\boldsymbol{ }\boldsymbol{\Delta }{\varvec{t}}\right)\boldsymbol{ }-\boldsymbol{ }{\varvec{r}}({\varvec{t}})}{\Delta t}$. Second, to eliminate the effect of noise, such as Brownian motion, on reorientation events, we smoothed the data by calculating running averages over 10 points, which corresponded to 50 ms intervals. Finally, given two data points, *r*(*t*) = [*x*(*t*), *y*(*t*)] and *r*(*t* + Δ*t*) = [*x*(*t* + Δ*t*), *y*(*t* + Δ*t*)], we defined the angle against the horizontal axis as *θ*(*t*). If two successive angle changes, *θ* (*t*_*1*_)–*θ* (*t*_*0*_) and *θ* (*t*_*2*_)–*θ* (*t*_*1*_), were over α, that point was identified as the end of the run. A new run began after three successive angle changes < α with a speed above 5 μm s^−1^; hence, the minimum duration of the run was 80 ms. The threshold α is described by the equation: α = c Δ*θ*_med_, where c is the coefficient and Δ*θ*_med_, the median directional change. We manually checked the trace and video to avoid false event detection and found that the best value of c was 3.

Using fluorescent-labeled cells, we constructed a kymograph at 2.5 ms intervals to measure swimming speed (Fig. 2). The flagellar rotation rate of each cell was measured by Fourier transform analysis (Fig. 2c, right). Under TIRF illumination, intensity changes were detected when fluorescent-labeled flagella contacted an evanescent field. Intensity changes in a 2 × 2 pixel grid were measured and calculated by fast Fourier transform analysis^27,28^.

## Supplementary information

Supplementary Information.

Supplementary Video 1.

Supplementary Video 2.

Supplementary Video 3.

Supplementary Video 4.

Supplementary Video 5.

Supplementary Video 6.

Supplementary Video 7.

Supplementary Video 8.

Supplementary Video 9.

Supplementary Video 10

Supplementary Video 11

Supplementary Video 12

Supplementary Video 13

## References

[1] Berg HC (2003). The rotary motor of bacterial flagella. Annu. Rev. Biochem..

[2] Miyata M (2020). Tree of motility—A proposed history of motility systems in the tree of life. Genes Cells.

[3] Sowa Y, Berry RM (2008). Bacterial flagellar motor. Q. Rev. Biophys..

[4] Berg HC, Anderson RA (1973). Bacteria swim by rotating their flagellar filaments. Nature.

[5] Silverman M, Simon M (1974). Flagellar rotation and the mechanism of bacterial motility. Nature.

[6] Berg HC, Brown DA (1972). Chemotaxis in *Escherichia coli* analysed by three-dimensional tracking. Nature.

[7] Turner L, Ryu WS, Berg HC (2000). Real-time imaging of fluorescent flagellar filaments. J. Bacteriol..

[8] Son, K., Guasto, J. S. & Stocker, R. Bacteria can exploit a flagellar buckling instability to change direction. *Nat. Phys.* **9**, 494, 10.1038/nphys2676. https://www.nature.com/articles/nphys2676#supplementary-information (2013).

[9] Xie L, Altindal T, Chattopadhyay S, Wu XL (2011). Bacterial flagellum as a propeller and as a rudder for efficient chemotaxis. Proc. Natl. Acad. Sci. USA..

[10] Murat D (2015). Opposite and coordinated rotation of amphitrichous flagella governs oriented swimming and reversals in a *Magnetotactic Spirillum*. J. Bacteriol..

[11] Kuhn MJ, Schmidt FK, Eckhardt B, Thormann KM (2017). Bacteria exploit a polymorphic instability of the flagellar filament to escape from traps. Proc. Natl. Acad. Sci. USA..

[12] Hintsche M (2017). A polar bundle of flagella can drive bacterial swimming by pushing, pulling, or coiling around the cell body. Sci. Rep..

[13] Kinosita Y, Kikuchi Y, Mikami N, Nakane D, Nishizaka T (2018). Unforeseen swimming and gliding mode of an insect gut symbiont, *Burkholderia* sp. RPE64, with wrapping of the flagella around its cell body. ISME J..

[14] Constantino MA (2018). Bipolar lophotrichous *Helicobacter suis* combine extended and wrapped flagella bundles to exhibit multiple modes of motility. Sci. Rep..

[15] Dunne KA (2017). Sequencing a piece of history: Complete genome sequence of the original *Escherichia coli* strain. Microbial Genom..

[16] Khetrapal V, Mehershahi KS, Chen SL (2017). Complete genome sequence of the original *Escherichia coli* isolate, Strain NCTC86. Genome Announc..

[17] Bachmann BJ (1972). Pedigrees of some mutant strains of *Escherichia coli* K-12. Bacteriol. Rev..

[18] Adler J (1973). A method for measuring chemotaxis and use of the method to determine optimum conditions for chemotaxis by *Escherichia coli*. J. Gen. Microbiol..

[19] Hazelbauer GL, Mesibov RE, Adler J (1969). *Escherichia coli* mutants defective in chemotaxis toward specific chemicals. Proc. Natl. Acad. Sci. U.S.A..

[20] Barker CS, Pruss BM, Matsumura P (2004). Increased motility of *Escherichia coli* by insertion sequence element integration into the regulatory region of the *flhD* operon. J. Bacteriol..

[21] Cremer J (2019). Chemotaxis as a navigation strategy to boost range expansion. Nature.

[22] Wolfe AJ, Berg HC (1989). Migration of bacteria in semisolid agar. Proc. Natl. Acad. Sci. USA..

[23] Taute KM, Gude S, Tans SJ, Shimizu TS (2015). High-throughput 3D tracking of bacteria on a standard phase contrast microscope. Nat. Commun..

[24] Macnab RM, Ornston MK (1977). Normal-to-curly flagellar transitions and their role in bacterial tumbling. Stabilization of an alternative quaternary structure by mechanical force. J. Mol. Biol..

[25] Kamiya R, Hotani H, Asakura S (1982). Polymorphic transition in bacterial flagella. Symp. Soc. Exp. Biol..

[26] Yoshioka K, Aizawa S, Yamaguchi S (1995). Flagellar filament structure and cell motility of *Salmonella typhimurium* mutants lacking part of the outer domain of flagellin. J. Bacteriol..

[27] Kinosita Y, Nishizaka T (2018). Cross-kymography analysis to simultaneously quantify the function and morphology of the archaellum. Biophys. Physicobiol..

[28] Kinosita Y, Uchida N, Nakane D, Nishizaka T (2016). Direct observation of rotation and steps of the archaellum in the swimming halophilic archaeon *Halobacterium salinarum*. Nat. Microbiol..

[29] Magariyama Y (1995). Simultaneous measurement of bacterial flagellar rotation rate and swimming speed. Biophys. J..

[30] Hotani H (1982). Micro-video study of moving bacterial flagellar filaments. III. Cyclic transformation induced by mechanical force. J. Mol. Biol..

[31] Kitao A (2006). Switch interactions control energy frustration and multiple flagellar filament structures. Proc. Natl. Acad. Sci. USA..

[32] Calladine CR (1975). Construction of bacterial flagella. Nature.

[33] Calladine CR, Luisi BF, Pratap JV (2013). A "mechanistic" explanation of the multiple helical forms adopted by bacterial flagellar filaments. J. Mol. Biol..

[34] Darnton NC, Berg HC (2007). Force-extension measurements on bacterial flagella: Triggering polymorphic transformations. Biophys. J..

[35] Hayashi F (2013). Key amino acid residues involved in the transitions of L- to R-type protofilaments of the *Salmonella* flagellar filament. J. Bacteriol..

[36] Wang C (2019). Role of flagellar hydrogen bonding in *Salmonella* motility and flagellar polymorphic transition. Mol. Microbiol..

[37] Wang W, Jiang Z, Westermann M, Ping L (2012). Three mutations in *Escherichia coli* that generate transformable functional flagella. J. Bacteriol..

[38] Kanto S, Okino H, Aizawa S, Yamaguchi S (1991). Amino acids responsible for flagellar shape are distributed in terminal regions of flagellin. J. Mol. Biol..

[39] Turner L, Zhang R, Darnton NC, Berg HC (2010). Visualization of Flagella during bacterial Swarming. J. Bacteriol..

[40] Mannik J, Driessen R, Galajda P, Keymer JE, Dekker C (2009). Bacterial growth and motility in sub-micron constrictions. Proc. Natl. Acad. Sci. U.S.A..

[41] Maki-Yonekura S, Yonekura K, Namba K (2010). Conformational change of flagellin for polymorphic supercoiling of the flagellar filament. Nat. Struct. Mol. Biol..

[42] Wang F (2017). A structural model of flagellar filament switching across multiple bacterial species. Nat. Commun..

[43] Yonekura K, Maki-Yonekura S, Namba K (2003). Complete atomic model of the bacterial flagellar filament by electron cryomicroscopy. Nature.

[44] Cisneros L, Dombrowski C, Goldstein RE, Kessler JO (2006). Reversal of bacterial locomotion at an obstacle. Phys. Rev. Stat. Nonlin. Soft Matter Phys..

[45] Fujii M, Shibata S, Aizawa S (2008). Polar, peritrichous, and lateral flagella belong to three distinguishable flagellar families. J. Mol. Biol..

[46] Taguchi F (2008). Effects of glycosylation on swimming ability and flagellar polymorphic transformation in *Pseudomonas syringae* pv tabaci 6605. J. Bacteriol..

[47] Armitage JP, Macnab RM (1987). Unidirectional, intermittent rotation of the flagellum of *Rhodobacter sphaeroides*. J. Bacteriol..

[48] Kuhn MJ (2018). Spatial arrangement of several flagellins within bacterial flagella improves motility in different environments. Nat. Commun..

[49] Echazarreta MA, Kepple JL, Yen LH, Chen Y, Klose KE (2018). A Critical region in the FlaA flagellin facilitates filament formation of the *Vibrio cholerae* flagellum. J. Bacteriol..

[50] Faulds-Pain A (2011). Flagellin redundancy in *Caulobacter crescentus* and its implications for flagellar filament assembly. J. Bacteriol..

[51] Wu L, Wang J, Tang P, Chen H, Gao H (2011). Genetic and molecular characterization of flagellar assembly in *Shewanella oneidensis*. PLoS ONE.

[52] Zhang H, Li L, Zhao Z, Peng D, Zhou X (2016). Polar flagella rotation in *Vibrio parahaemolyticus* confers resistance to bacteriophage infection. Sci. Rep..

[53] Liu B (2014). Helical motion of the cell body enhances *Caulobacter crescentus* motility. Proc. Natl. Acad. Sci. U.S.A..

[54] Mohari B (2015). Novel pseudotaxis mechanisms improve migration of straight-swimming bacterial mutants through a porous environment. mBio.

[55] Sporing I (2018). Hook length of the bacterial flagellum is optimized for maximal stability of the flagellar bundle. PLoS Biol..

[56] Kinosita Y (2020). Distinct chemotactic behavior in the original *Escherichia coli* K-12 depending on forward-and-backward swimming, not on run-tumble movements. BioRxiv.

[57] Datsenko KA, Wanner BL (2000). One-step inactivation of chromosomal genes in *Escherichia coli* K-12 using PCR products. Proc. Natl. Acad. Sci. U.S.A..

[58] Bochner BR, Huang HC, Schieven GL, Ames BN (1980). Positive selection for loss of tetracycline resistance. J. Bacteriol..

[59] Kinosita Y, Miyata M, Nishizaka T (2018). Linear motor driven-rotary motion of a membrane-permeabilized ghost in *Mycoplasma mobile*. Sci. Rep..

[60] Kinosita Y (2014). Unitary step of gliding machinery in *Mycoplasma mobile*. Proc. Natl. Acad. Sci. U.S.A..

[61] Nikata T, Sumida K, Kato J, Ohtake H (1992). Rapid method for analyzing bacterial behavioral responses to chemical stimuli. Appl. Environ. Microbiol..

[62] Ishida T (2019). Sodium-powered stators of the bacterial flagellar motor can generate torque in the presence of phenamil with mutations near the peptidoglycan-binding region. Mol. Microbiol..

