# Supplementary material for: Distinct chemotactic behavior in the original Escherichia coli K-12 depending on forward-and-backward swimming, not on run-tumble movements
Source: Sci Rep. 2020 Sep 28;10:15887. doi: 10.1038/s41598-020-72429-1 (PMC7522084; doi:10.1038/s41598-020-72429-1)
Supplement: Supplementary file 1 — Supplementary Information. [file 41598_2020_72429_MOESM1_ESM.pdf]

Distinct chemotactic behavior in the original *Escherichia coli* K-12 depending on forward-and-backward swimming, not on run-tumble movements

Yoshiaki Kinosita[1,2, ¶,*], Tsubasa Ishida[3], Myu Yoshida[3], Rie Ito[3], Yusuke V. Morimoto[4], Kazuki Goto[1], Richard M. Berry[2], Takayuki Nishizaka[1] & Yoshiyuki Sowa[3,*]

[1]Department of Physics, Gakushuin University, 1-5-1 Mejiro, Toshima-ku, Tokyo 171-8588, Japan.

[2]Department of Physics, University of Oxford, Park load OX1 3PU, Oxford, UK

[3]Department of Frontier Bioscience and Research Center for Micro-Nano Technology, Hosei University, Tokyo 184-8584, Japan

[4]Department of Physics and Information Technology, Faculty of Computer Science and Systems Engineering, Kyushu Institute of Technology, Iizuka, Fukuoka, Japan

[¶]Present address: Molecular Physiology Laboratory, RIKEN, Japan

*Correspondence should be addressed to yoshiaki.kinosita@gmail.com or ysowa@hosei.ac.jp

This file includes:
Supplementary Figures 1-10
Supplementary Table 1-3
Captions for Supplementary Videos
Supplementary References

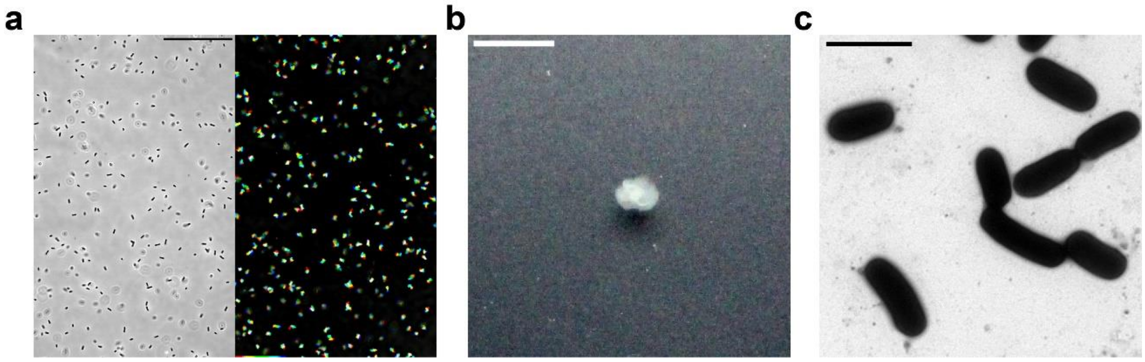

Supplementary Figure 1 Characterization of swimming motility and structural parameters of SHU101

(a) *Left*: Phase-contrast image of the non-flagellated mutant, SHU101. Scale bar, 50 μm. *Right*: The sequential phase-contrast images with 165-ms intervals were integrated for 5 s with the intermittent color code ‘red, → yellow, → green, → cyan, → blue.’ The processive linear movements could not be seen, indicating that the SHU101 is a non-motile strain. (b) Motility of SHU101 cells on a 0.25 % (wt/vol) soft-agar plate at 30ºC for 7 h. Scale bar, 0.5 cm. (c) Electron micrograph. Scale bar, 2 μm.

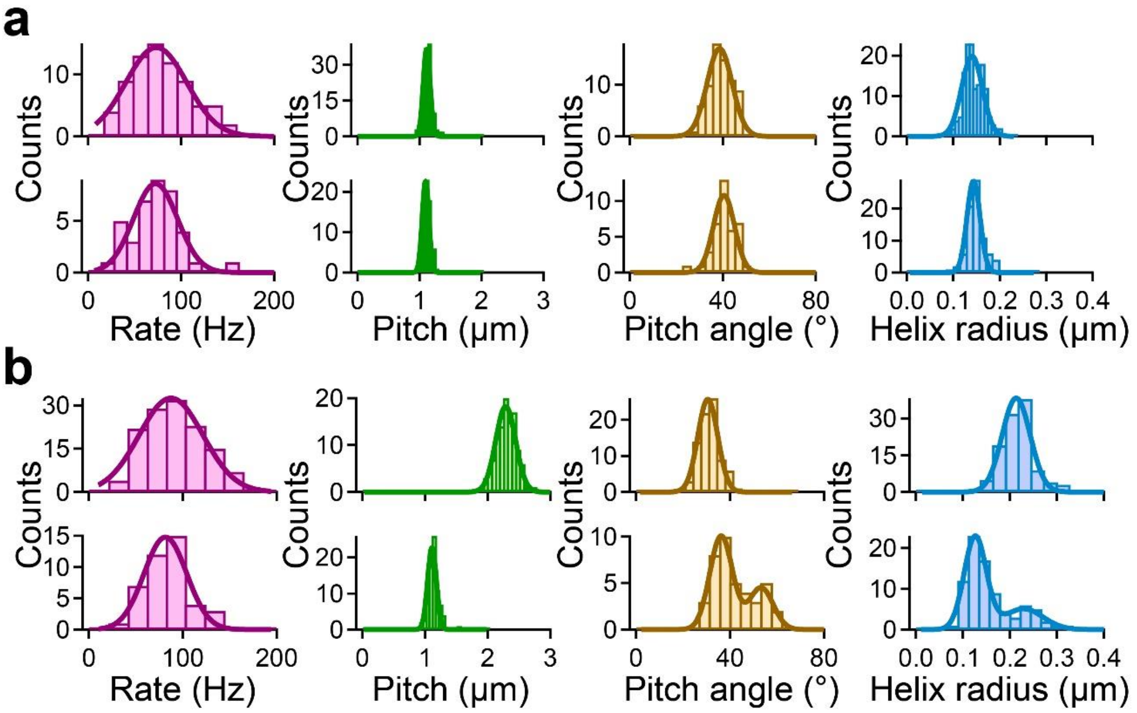

Supplementary Figure 2 Quantification of rotational rate and structural parameters of flagella under total internal reflection fluorescence microscope (TIRFM)

(a) Histograms of the rotation rate and structural parameters during CCW (*top*) and CW rotation (*bottom*) in ATCC10798 cells. Solid line represents the Gaussian fitting. The peaks and SDs of rotational rates were 73.3 ± 32.8 Hz in CCW direction (n = 74) and 72.4 ± 23.7 Hz in CW direction (n = 39). The flagellar pitches were 1.1 ± 0.1 μm in CCW (n =142) and 1.1 ± 0.1 μm in CW direction (n = 80). The pitch angles were 38.6 ± 5.4 degree in CCW (n = 70) and 40.5 ± 4.5 degree in CW (n = 39). The helix radii were 0.14 ± 0.02 μm in CCW (n = 142) and 0.14 ± 0.01 μm in CW (n = 80). (b) Histograms of the structure and rotation rate of flagella of W3110 during left-handed (*top*) and right-handed (*bottom*) state. The rotation rates were 87.6 ± 34.0 Hz under a left-handed state (n = 133) and 81.5 ± 22.7 Hz under a right-handed state (n = 42). The flagellar pitches were 2.3 ± 0.2 μm under a left-handed state (n = 112) and 1.1 ± 0.1 μm under a right-handed state (n = 84). The pitch angles were 30.5 ± 4.2 degree under a left-handed state (n = 81); 36.2 ± 4.8 and 53.2 ± 5.1 degree under a right-handed state (n = 40). The helix radii were 0.21 ± 0.03 μm under a left-handed state (n = 112); 0.13 ± 0.02 μm and 0.23 ± 0.04 μm under a right-handed state (n = 84).

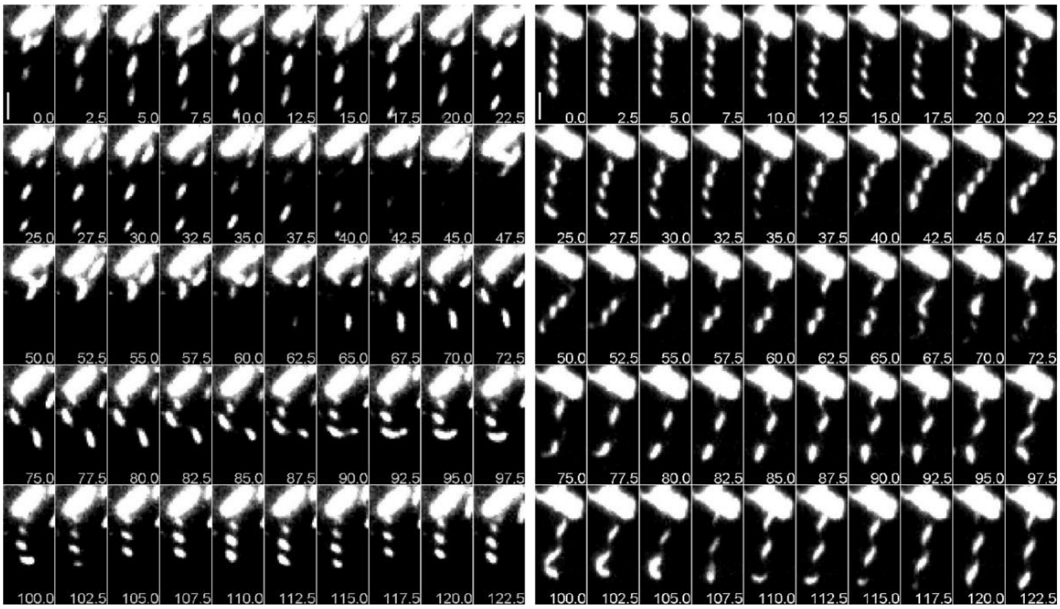

Supplementary Figure 3 Real-time imaging of flagellar polymorphism in W3110

Sequential images of the flagellar polymorphism at 2.5-ms intervals. *Left*: The orientation of flagellar filament(s) was from the first quadrant to the third quadrant relative to the major axis of the filament, indicating that the helicity of filaments was left-handed. From 0 to 22.5 ms, the wave of flagella propagated in a direction away from the hook end toward the flagellar tip, indicating that the flagella rotated in CCW direction. The direction of rotation was switched at 30.0 ms, and then their helicity changed from the left-handed into right-handed within 50 ms. Taken together that the pitch angle of flagellar filaments was 58 degrees, the filament form was the semi-coiled. Scale bar, 2 μm. *Right*: The orientation of flagellar filament (s) was from the second quadrant to the fourth quadrant relative to the major axis of the filament, indicating that the helicity of filaments was right-handed. We concluded that the flagellar form was the curly state with the fact of the 40°-pitch angle. From 0 to 17.5 ms, the flagellar filament did not move. At 20 ms, the flagellar filaments gradually moved, suggesting that the flagella started to rotate. The flagellar filaments dynamically twisted at 40 ms, and then the right-handed and left-handed flagellar were combined into a single filament at 67.5 and 105.0 ms, which was also seen in another bacterium. Finally, the curry filament(s) transformed into the normal state within 100 ms. Scale bar, 2 μm. Data from Supplementary Videos 6 and 7.

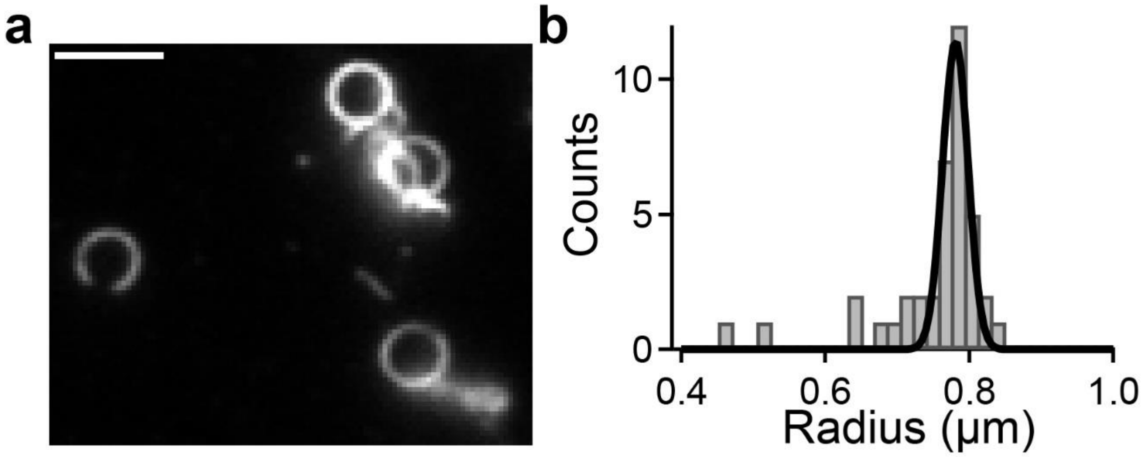

Supplementary Figure 4 Quantification of flagellar radius at coiled state in W3110

(a) The fluorescent micrograph of coiled-state flagellar filaments. Scale bar; 3 μm. (b) Histogram of the radius of coiled flagella. The solid line represents the Gaussian fitting, where the peak and SD are 0.78 ± 0.02 μm (n = 39).

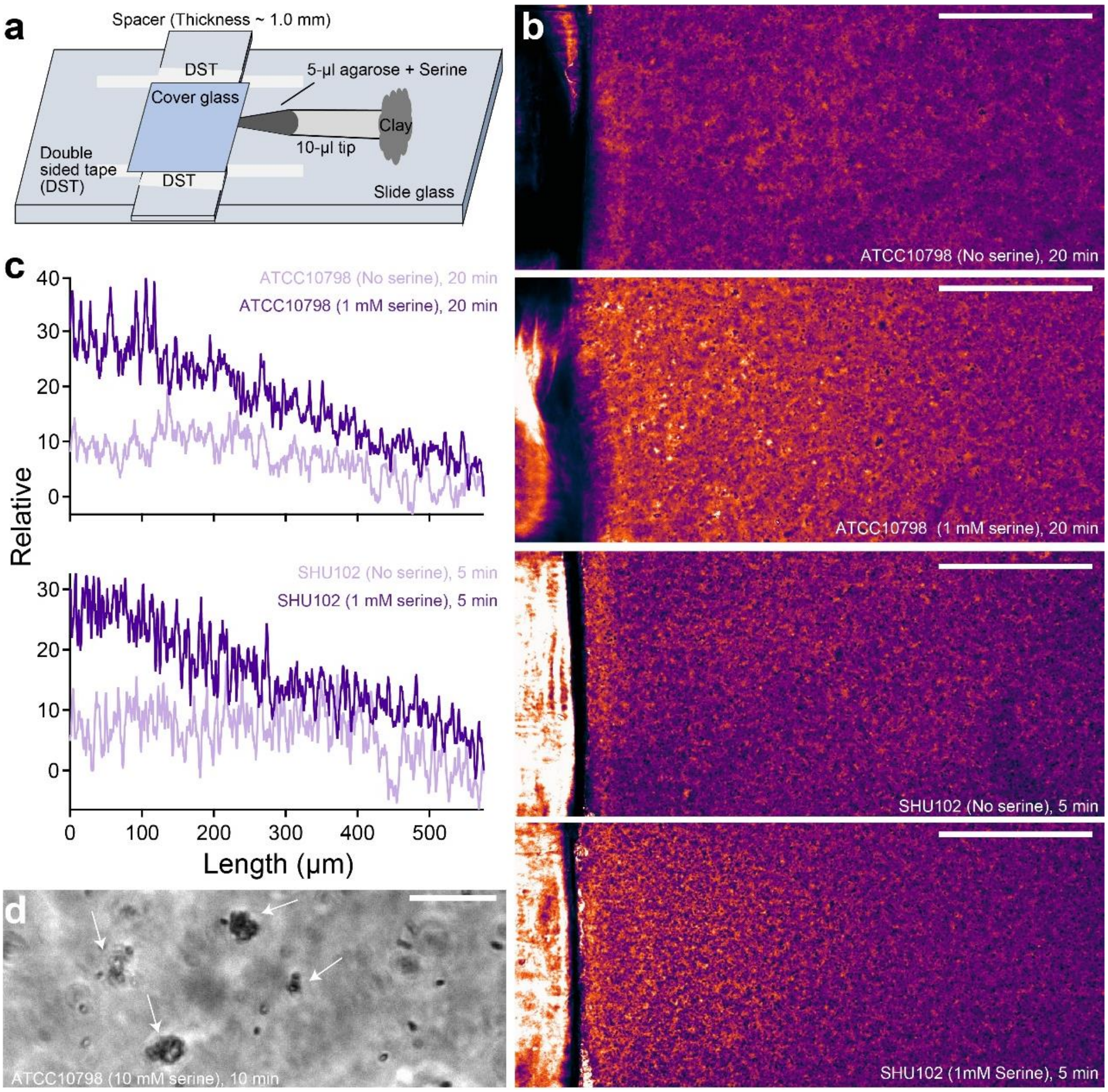

Supplementary Figure 5 Chemotactic response of ATCC10798 [*fliC*(N87K)] and SHU102 [*fliC*(N87K)::*fliC*] cells

(a) The schematic of the tip (capillary) assay. The tip contains a 5-μl buffer containing 1 % (wt/vol) agarose, and the other end was sealed with cray to avoid an effect of oxygen on a chemotactic response. (b) Pseudo-colors of phase-contrast images of ATCC10798 (*top*) and SHU102 cells (*bottom*). In the presence of 1 mM serine, both *E.coli* strains exhibit a chemotactic response and gather near a tip (orange color). Scale bar, 200 μm. (c) Intensity profiles of b. (d) A phase-contrast image of ATCC10798 cells in the presence of 10 mM serine. In the presence of serine, ATCC10798 cells tend to gather and aggregate each other, which was not detected in SHU102 cells. Scale bar, 20 μm.

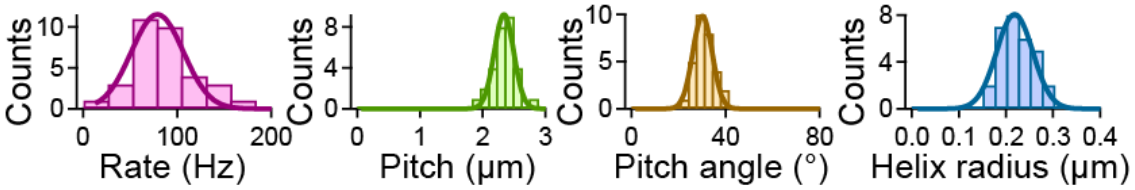

Supplementary Figure 6 Quantification of rotational rate and structural parameters of flagella of SHU102 under TIRFM

Histograms of the structure and rotation rate of flagella of SHU102. The solid line represents the Gaussian fitting. Peaks and SDs of the rotation rate were 78.9 ± 27.5 Hz (n = 33). The flagellar pitch was 2.3 ± 0.2 μm (n = 30). The pitch angles were 30.2 ± 4.1 degrees (n = 30). The helix radius was 0.22 ± 0.04 μm (n = 30).

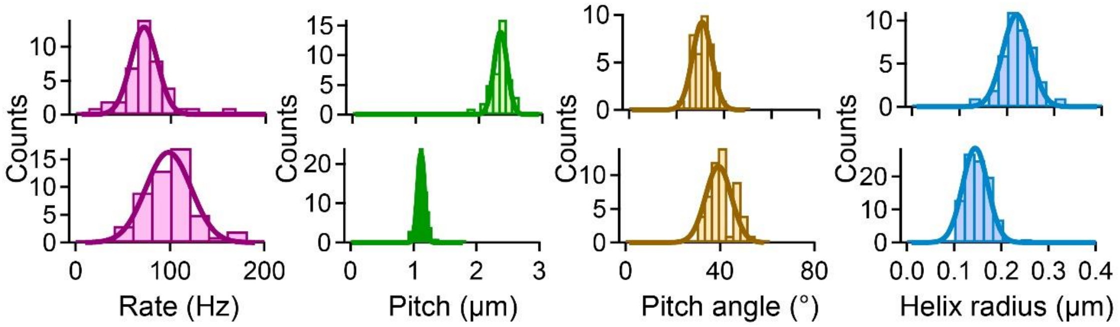

Supplementary Figure 7 Quantification of rotational rate and structural parameters of HCB1336/pYS10 and HCB1336/pSHU61 under TIRFM

Histograms of the structural properties and rotation rate of flagella of *E. coli* HCB1336 (Δ*fliC* strain) carrying the plasmid encoding wild-type *fliC* (pYS10) and *fliC*(N87K) (pSHU61). Solid line represents the Gaussian fitting. Peaks and SDs of the rotation rate were 71.8 ± 14.1 Hz in HCB1336/pYS10 (*top*, n = 41) and 98.4 ± 23.9 Hz in HCB1336/pSHU61 cells (*bottom*, n = 50) and. The flagellar pitches were 2.3 ± 0.1μm in HCB1336/pYS10 cells (*top*, n = 40) and 1.1 ± 0.1 μm in HCB1336/pSHU61 cells (*bottom*, n = 94). The pitch angles were 30.8 ± 4.1 degree in HCB1336/pYS10 cells (*top*, n = 37) and 39.3 ± 5.5 degree in pSHU61/HCB1336 cells (*bottom*, n = 52). The helix radii were 0.22 ± 0.03 μm in HCB1336/pYS10 cells (*top*, n = 40) and 0.14 ± 0.03 μm in HCB133/pSHU61 cells (*bottom*, n = 94).

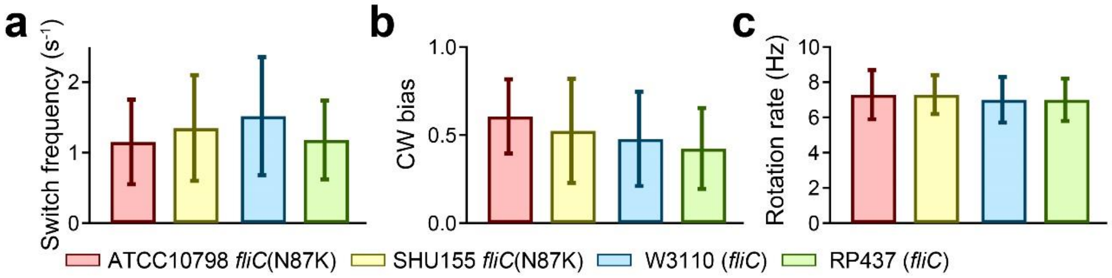

Supplementary Figure 8 Quantification of motor properties by tethered-cell assay

(a) Switching frequency between *E. coli* strains for 10 sec. The average and SD were 1.15 ± 0.60 s^{-1} in ATCC10798 (n = 99), 1.35 ± 0.75 s^{-1} in SHU155 (n = 67), 1.52 ± 0.84 s^{-1} in W3110 (n = 53), and 1.18 ± 0.56 s^{-1} in RP437 (n = 49). (b) CW bias ($Time_{CW}/Time_{Total}$). The average and SD were 0.61 ± 0.21 in ATCC10798 (n = 99) and 0.52 ± 0.30 in SHU155 (n = 67), 0.48 ± 0.27 in W3110 (n = 53), and 0.42 ± 0.23 in RP437 (n = 49). (c) Rotation rates. The average and SD were 7.3 ± 1.4 Hz in ATCC10798 (n = 99), 7.3 ± 1.1 Hz in SHU155 (n = 67), 7.0 ± 1.3 Hz in W3110 (n = 53), and 7.0 ± 1.2 Hz in RP437 (n = 49). ATCC10798 and W3110 data from Figure 3.

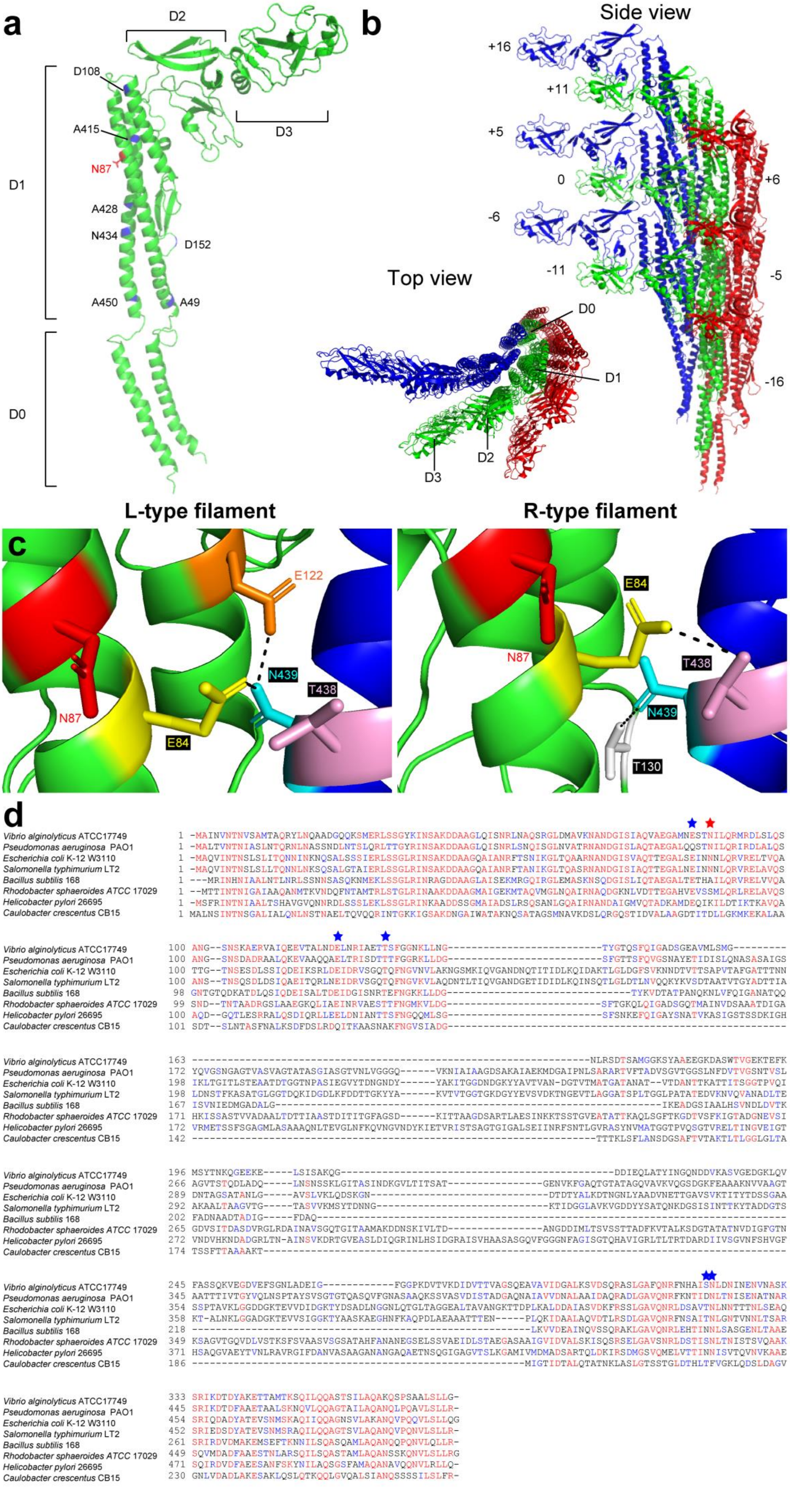
a
D2
D3
D1
D0
D108
A415
N87
A428
N434
D152
A450
A49
b
Side view
+16
+11
+5
0
-6
-11
+6
-5
-16
Top view
D0
D1
D2
D3
L-type filament
R-type filament
c
E122
N439
T438
N87
E84
E84
N87
T438
N439
T130
d
Vibrio alginolyticus ATCC17749 1 -MAINVNTNVSAMTAQRYLNQAADGQQKSMERLSSGYKINSAKDDAAGLQISNRLNAQSRGLDMAVKNANDGISIAQVAEGAMNESTNILQRMRDLSLQS
Pseudomonas aeruginosa PAO1 1 -MALTVNTNIASLNTQRNLNASSNDLNTSLQRLTTGYRINSAKDDAAGLQISNRLSNQISGLNVATRNANDGISLAQTAEGALQQSTNILQRIRDLALQS
Escherichia coli K-12 W3110 1 -MAQVINTNSLSLITQNNINKNQSALSSSIERLSSGLRINSAKDDAAGQAIANRFTSNIKGLTQAARNANDGISVAQTTEGALSEINNNLQRVRELTVQA
Salomonella typhimurium LT2 1 -MAQVINTNSLSLLTQNNLNKSQSALGTAIERLSSGLRINSAKDDAAGQAIANRFTANIKGLTQASRNANDGISIAQTTEGALNEINNNLQRVRELAVQS
Bacillus subtilis 168 1 ---MRINHNIAALNTLNRLSSNNSASQKNMEKLSSGLRINRAGDDAAGLAISEKMRGQIRGLEMASKNSQDGISLIQTAEGALTETHAILQRVRELVVQA
Rhodobacter sphaeroides ATCC 17029 1 --MTTINTNIGAIAAQANMTKVNDQFNTAMTRLSTGLRINAAKDDAAGMAIGEKMTAQVMGLNQAIRNAQDGKNLVDTTEGAHVEVSSMLQRLRELAVQS
Helicobacter pylori 26695 1 -MSFRINTNIAALTSHAVGVQNNRDLSSSLEKLSSGLRINKAADDSSGMAIADSLRSQSANLGQAIRNANDAIGMVQTADKAMDEQIKILDTIKTKAVQA
Caulobacter crescentus CB15 1 MALNSINTNSGALIALQNLNSTNAELTQVQQRINTGKKIGSAKDNGAIWATAKNQSATAGSMNAVKDSLQRGQSTIDVALAAGDTITDLLGKMKEKALAA
Vibrio alginolyticus ATCC17749 100 ANG--SNSKAERVAIQEEVTALNDELNRIAETTSFGGNKLLNG-------------------------TYGTQSFQIGADSGEAVMLSMG---------
Pseudomonas aeruginosa PAO1 100 ANG--SNSDADRAALQKEVAAQQAELTRISDTTTFGGRKLLDG-------------------------SFGTTSFQVGSNAYETIDISLQNASASAIGS
Escherichia coli K-12 W3110 100 TTG--TNSESDLSSIQDEIKSRLDEIDRVSGQTQFNGVNVLAKNGSMKIQVGANDNQTITIDLKQIDAKTLGLDGFSVKNNDTVTTSAPVTAFGATTTNN
Salomonella typhimurium LT2 100 ANS--TNSQSDLDSIQAEITQRLNEIDRVSGQTQFNGVKVLAQDNTLTIQVGANDGETIDIDLKQINSQTLGLDTLNVQQKYKVSDTAATVTGYADTTIA
Bacillus subtilis 168 98 GNTGTQDKATDLQSIQDEISALTDEIDGISNRTEFNGKKLLDG------------------------------TYKVDTATPANQKNLVFQIGANATQQ
Rhodobacter sphaeroides ATCC 17029 99 SND--TNTAADRGSLAAEGKQLIAEINRVAESTTFNGMKVLDG-------------------------SFTGKQLQIGADSGQTMAINVDSAAATDIGA
Helicobacter pylori 26695 100 AQD--GQTLESRRALQSDIQRLLEELDNIANTTSFNGQQMLSG-------------------------SFSNKEFQIGAYSNATVKASIGSTSSDKIGH
Caulobacter crescentus CB15 101 SDT--SLNTASFNALKSDFDSLRDQITKAASNAKFNGVSIADG---
Vibrio alginolyticus ATCC17749 163 --NLRSDTSAMGGKSYAAEEGKDASWTVGEKTEFK
Pseudomonas aeruginosa PAO1 172 YQVGSNGAGTVASVAGTATASGIASGTVNLVGGGQ------VKNIAIAAGDSAKAIAEKMDGAIPNLSARARTVFTADVSGVTGGSLNFDVTVGSNTVSL
Escherichia coli K-12 W3110 198 IKLTGITLSTEAATDTGGTNPASIEGVYTDNGNDY------YAKITGGDNDGKYYAVTVAN-DGTVTMATGATANAT--VTDANTTKATTITSGGTPVQI
Salomonella typhimurium LT2 198 LDNSTFKASATGLGGTDQKIDGDLKFDDTTGKYYA------KVTVTGGTGKDGYYEVSVDKTNGEVTLAGGATSPLTGGLPATATEDVKNVQVANADLTE
Bacillus subtilis 168 167 ISVNIEDMGADALG---IKEADGSIAALHSVNDLDVTK
Rhodobacter sphaeroides ATCC 17029 171 HKISSASTVVADAALTDTTIAASTDITITGFAGSD------KITTAAGDSARTLAESINKKTSSTGVEATATTKAQLSGFTKGDTVSFKIGTADGNEVSI
Helicobacter pylori 26695 172 VRMETSSFSGAGMLASAAAQNLTEVGLNFKQVNGVNDYKIETVRISTSAGTGIGALSEIINRFSNTLGVRASYNVMATGGTPVQSGTVRELTINGVEIGT
Caulobacter crescentus CB15 142 --TTTKLSFLANSDGSAFTVTAKTLTLGGLGLTA
Vibrio alginolyticus ATCC17749 196 MSYTNKQGEEKE-----LSISAKQG--DDIEQLATYINGQNDDVKASVGEDGKLQV
Pseudomonas aeruginosa PAO1 266 AGVTSTQDLADQ-----LNSNSSKLGITASINDKGVLTITSAT----------------GENVKFGAQTGTATAGQVAVKVQGSDGKFEAAAKNVVAAGT
Escherichia coli K-12 W3110 289 DNTAGSATANLG-----AVSLVKLQDSKGN-----------------------------DTDTYALKDTNGNLYAADVNETTGAVSVKTITYTDSSGAA
Salomonella typhimurium LT2 292 AKAALTAAGVTG-----TASVVKMSYTDNNG----------------------------KTIDGGLAVKVGDDYYSATQNKDGSISINTTKYTADDGTS
Bacillus subtilis 168 202 FADNAADTADIG-----FDAQ--
Rhodobacter sphaeroides ATCC 17029 265 GDVSITDASDVRGLRDAINAVSGQTGITAAMAKDDNSKIVLTD----------------ANGDDIMLTSVSSTTADFKVTALKSDGTATATNVDIGFGTN
Helicobacter pylori 26695 272 VNDVHKNDADGRLTN-AINSVKDRTGVEASLDIQGRINLHSIDGRAISVHAASASGQVFGGGNFAGISGTQHAVIGRLTLTRTDARDIIVSGVNFSHVGF
Caulobacter crescentus CB15 174 TSSFTTAAAAKT---
Vibrio alginolyticus ATCC17749 245 FASSQKVEGDVEFSGNLADEIG------------FGGPKDVTVKDIDVTTVAGSQEAVAVIDGALKSVDSQRASLGAFQNRFNHAISNLDNINENVNASK
Pseudomonas aeruginosa PAO1 345 AATTTIVTGYVQLNSPTAYSVSGTGTQASQVFGNASAAQKSSVASVDISTADGAQNAIAVVDNALAAIDAQRADLGAVQNRFKNTIDNLTNISENATNAR
Escherichia coli K-12 W3110 354 SSPTAVKLGGDDGKTEVVDIDGKTYDSADLNGGNLQTGLTAGGEALTAVANGKTTDPLKALDDAIASVDKFRSSLGAVQNRLDSAVTNLNNTTTNLSEAQ
Salomonella typhimurium LT2 358 KT-ALNKLGGADGKTEVVSIGGKTYAASKAEGHNFKAQPDLAEAAATTTEN-----PLQKIDAALAQVDTLRSDLGAVQNRFNSAITNLGNTVNNLTSAR
Bacillus subtilis 168 218 ---LKVVDEAINQVSSQRAKLGAVQNRLEHTINNLSASGENLTAAE
Rhodobacter sphaeroides ATCC 17029 349 KSAGVTGQVDLVSTKSFSVAASVSGSATAHFANANEGSELSSVAEIDLSTAEGASAAIGVIDVALSKISQSRSELGAVSNRLDSTISNLTNISTSVQAAK
Helicobacter pylori 26695 371 HSAQGVAEYTVNLRAVRGIFDANVASAAGANANGAQAETNSQGIGAGVTSLKGAMIVMDMADSARTQLDKIRSDMGSVQMELVTTINNISVTQVNVKAAE
Caulobacter crescentus CB15 186 ---MIGTIDTALQTATNKLASLGTSSTGLDTHLTFVGKLQDSLDAGV
Vibrio alginolyticus ATCC17749 333 SRIKDTDYAKETTAMTKSQILQQASTSILAQAKQSPSAALSLLG-
Pseudomonas aeruginosa PAO1 445 SRIKDTDFAAETAALSKNQVLQQAGTAILAQANQLPQAVLSLLR-
Escherichia coli K-12 W3110 454 SRIQDADYATEVSNMSKAQIIQQAGNSVLAKANQVPQQVLSLLQG
Salomonella typhimurium LT2 452 SRIEDSDYATEVSNMSRAQILQQAGTSVLAQANQVPQNVLSLLR-
Bacillus subtilis 168 261 SRIRDVDMAKEMSEFTKNNILSQASQAMLAQANQQPQNVLQLLR-
Rhodobacter sphaeroides ATCC 17029 449 SQVMDADFAAESTNLARSQILSQASTAMLAQANSSKQNVLSLLRG
Helicobacter pylori 26695 471 SQIRDVDFAEESANFSKYNILAQSGSFAMAQANAVQQNVLRLLQ-
Caulobacter crescentus CB15 230 GNLVDADLAKESAKLQSLQTKQQLGVQALSIANQSSSSILSLFR-

Supplementary Figure 9 Structure and sequence of flagellin

(a) Subunit structure of L-type flagellar subunit (PDB: 3A5X). The important residues for a curly filament are shown. (b) Side views of L-type flagellar filament. The number of protofilaments is shown. *Inset*: Top view of filaments. (c) L-type (*left*, PDB: 3A5X) and R-type (*right*, PDB: 1UCU) straight filaments of *S. typhimurium* are shown [3,4]. Hydrogen bonds interaction (black) between S0 (green) and S5 subunits (blue). E84, N87, E122, T130 of the S0 subunit are colored in yellow, red, orange, and white, respectively; T438 and N439 of the S5 subunit are colored in cyan and pink, respectively. (d) Alignments of the flagellin amino acid sequence from typical bacteria. The red star represents N87 residue; blue stars indicate E84, E122, T130, T438, N439 residues. Images were generated using PyMOL 2.4 (https://pymol.org/2/).

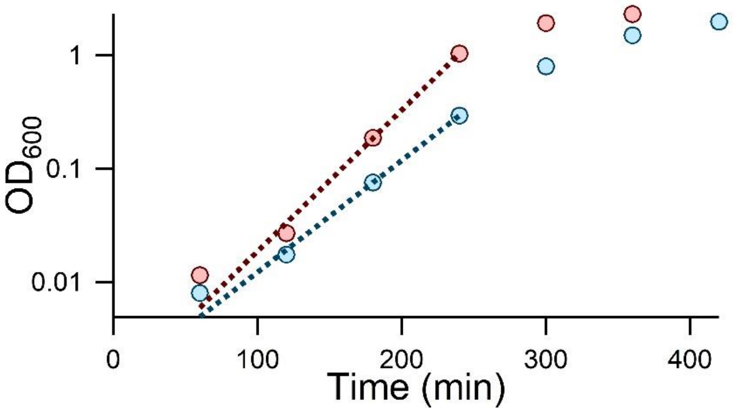

Supplementary Figure 10 Growth curve in *Escherichia coli* K-12 ATCC10798 and W3110

The single colony was scrutinized by the tip, resuspended into 50-ml LB medium, and then cultured at 37°C with shaking. The absorbance of cells at 600 nm were periodically measured with 60-min intervals. During the exponential phase, we fitted the data as the following equation: $f(t) = A \times 2^{\frac{t}{tau}}$, where *A* the constant and *tau* the doubling time. The doubling time was estimated to be ~24 min in ATCC10798 (red) and 30 min in W3110 (blue). The experiments were performed two times.

Supplementary Table 1

Structural parameters and kinematics of the cell body and flagella in *Escherichia coli*. Values were directly measured by either a transmission electron microscopy (EM) or optical microscopy (OM).

	EM						OM	
Cells	Body length (μm)	Body width (μm)	Flagellar length (μm)	Flagellar helix radius (μm)	Flagellar number	Flagellar pitch (μm)	Swimming speed (μm s^{-1})	Rotation rate (Hz)
ATCC10798	1.9±0.3 (62)	0.70±0.07 (62)	4.7±1.1 (62)	0.14±0.03 (59)	1.8±0.6 (61)	1.3±0.2 (59)	13.2±4.4 (70)	73.3±32.8 (74)
W3110	2.1±0.5 (50)	0.77±0.13 (50)	7.3±1.9 (48)	0.22±0.05 (42)	6.5±2.3 (39)	3.0±0.2 (41)	32.5±6.6 (50)	87.6±34.0 (133)
pYS10/HCB1336	2.1±0.4 (57)	0.83±0.17 (57)	5.8±1.6 (57)	0.22±0.06 (44)	3.2±1.3 (57)	2.7±0.3 (42)	21.7±4.5 (53)	71.8±14.1 (41)
pSHU61/HCB1336	1.8±0.2 (46)	0.73±0.12 (46)	4.5±0.9 (44)	0.14±0.03 (46)	3.1±1.3 (46)	1.4±0.1 (46)	12.0±4.2 (53)	98.4±23.9 (50)
SHU101	1.9±0.3 (21)	0.74±0.06 (21)	n.d.	n.d.	0 (21)	n.d.	n.d.	n.d.
SHU102	2.1±0.3 (32)	0.95±0.20 (32)	6.9±1.5 (29)	0.20±0.04 (27)	5.3±2.4 (31)	2.5±0.2 (27)	26.3±6.0 (45)	78.9±27.5 (33)

Supplementary Table 2. Strains and plasmids

Strain/plasmid	Relevant Phenotype/ Genotype	Source of Reference
Strains		
**W3110**	Wild type	
**ATCC10798**	Wild type (*fliC* N87K)	This study
**RP437**	Wild type for chemotaxis	[5]
**HCB1336**	CM735 Δ*fliC*	H. C. Berg
**SHU101**	ATCC10798 *fliC*(N87K)::*tetRA*	This study
**SHU102**	ATCC10798 *fliC*(N87K)::*fliC*	This study
**SHU155**	W3110 *fliC*::*fliC*(N87K)	This study
Plasmids		
**pKD46**	Red system	[6]
**pYS10**	FliC expression plasmid; pBR322 derivative	[7]
**pSHU61**	FliC(N87K) expression plasmid; pBR322 derivative	This study

Supplementary Table 3. Primers

Name	5' > 3'	Note
**FliC ATCC FW**	GGAAACCCAATACGTAATCA	Forward primer for PCR and sequencing of *fliC* in ATCC10798
**FliC ATCC Rev**	CAATTTGGCGTTGCCGTCAGTCTC	Reverse primer for PCR and sequencing of *fliC* in ATCC10798
**1217_fliC(N87K)-f(QC)**	CTGTCCGAAATCAACAAGAACTTACAGCGTGTG	Forward primer for mutagenesis on *fliC*
**1218_fliC(N87K)-r(QC)**	CACACGCTGTAAGTTCTTGTTGATTTCGGACAG	Reverse primer for mutagenesis on *fliC*
**0196_fliC-tetRA-F**	CAATATAGGATAACGAATCATGGCACAAGTCATTAATACCTTAAGACCCACTTTCACATTT	Forward primer for PCR of *tetRA* cassette
**0197_fliC-tetRA-R**	ACCCTGCAGCAGAGACAGAACCTGCTGCGGTACCTGGTTACTAAGCACTTGTCTCCTG	Reverse primer for PCR of *tetRA* cassette
**1232_fliC-F**	CAATATAGGATAACGAATCATGGCACAAGT	Forward primer for PCR of wild-type *fliC* cassette
**0199_fliC-R**	TTAACCCTGCAGCAGAGACAGA	Reverse primer for PCR of wild-type *fliC* cassette and sequencing of *fliC*
**0210_tetRA-785-R**	GGCAAGACTGGCATGATAAGGCC	Reverse primer for Colony PCR
**0211_tetRA-1090-F**	GTGAAGTGGTTCGGTTGGTTAGGG	Forward primer for Colony PCR
**0219_fliC-(-175)-F**	ATAGCGGGAATAAGGGGCAGA	Forward primer for Colony PCR and sequencing of *fliC*
**0220_fliC- (+250)-R**	GGTGGCGGGGAAGCACGTTGC	Reverse primer for Colony PCR and sequencing of *fliC*
**0198__fliC-F**	ATGGCACAAGTCATTAATACC	Forward primer for sequencing of *fliC*

Captions for Supplementary Videos

Supplementary Video 1
Swimming motility of *E. coli* K-12 ATCC10798 (*left*) and *E. coli* K-12 W3110 (*right*) observed under a phase-contrast microscope. ATCC10798 cells show 180°-reversals against to original swimming direction with the cell body rolling, whereas W3110 cells swim with the run and tumble movements. Scale bar, 20 μm.

Supplementary Video 2
Swimming motility of ATCC10798 (*left*) and W3110 (*right*) under a conventional fluorescent microscope. Scale bar, 20 μm.

Supplementary Video 3
High-speed imaging of flagellar rotation of ATCC10798 (*left*) and W3110 (*right*) using an EMCCD camera under a fluorescent microscope. Scale bar, 5 μm.

Supplementary Video 4
Flagellar rotation of ATCC10798 under TIRFM. Scale bar, 4 μm.

Supplementary Video 5
Flagellar rotation of W3110 under TIRFM. Scale bar, 5 μm.

Supplementary Video 6
Real-time imaging of a polymorphic flagellar change under TIRFM. A rotational direction was changed at 0.11 sec; subsequently, a normal left-handed filament was transformed into the right-handed semi-coiled filament. Scale bar, 2 μm.

Supplementary Video 7
Real-time imaging of a polymorphic flagellar change under TIRFM. A rotational direction was changed at 0.17 sec; subsequently, a right-handed curly filament was transformed into the normal left-handed filament. Scale bar, 2 μm.

Supplementary Video 8
Swimming motility of SHU102 (*left*), HCB1336/pYS10 (*middle*), and HCB1336/pSHU61 cells (*right*) under a phase-contrast microscope. Scale bar, 50 μm.

Supplementary Video 9

Swimming motility of SHU102 cells under a conventional fluorescent microscope. Scale bar, 20 μm.

Supplementary Video 10

Chemotactic response of ATCC10798 (*top*) and SHU102 cells (*bottom*) under a phase-contrast microscope. For the first 10 sec, cells swim in the absence of serine. For the last 10 sec, both cells swim in the presence of 1 mM serine. Scale bar, 200 μm.

Supplementary Video 11

Flagellar rotation of SHU102 (*left*), HCB1336/pYS10 (*middle*), and HCB1336/pSHU61 cells (*right*) under TIRFM. Scale bar, 2 μm.

Supplementary Video 12

Swimming motility of ATCC10798 (*left*) and W3110 (*right*) on a 0.2 % agarose pad. ATCC10798 cells cannot migrate after stuck, whereas W3110 cells keep moving with 180°-reversals to escape from a stuck. Scale bar, 50 μm.

Supplementary Video 13

Swimming motility of ATCC10798 (*left*) and W3110 (*right*) in the presence of 15 % ficoll. Scale bar, 20 μm.

Supplementary References

1. Taute, K. M., Gude, S., Tans, S. J. & Shimizu, T. S. High-throughput 3D tracking of bacteria on a standard phase contrast microscope. *Nature communications* **6**, 8776, doi:10.1038/ncomms9776 (2015).
2. Kamiya, R., Hotani, H. & Asakura, S. Polymorphic transition in bacterial flagella. *Symposia of the Society for Experimental Biology* **35**, 53-76 (1982).
3. Maki-Yonekura, S., Yonekura, K. & Namba, K. Conformational change of flagellin for polymorphic supercoiling of the flagellar filament. *Nature structural & molecular biology* **17**, 417-422, doi:10.1038/nsmb.1774 (2010).
4. Yonekura, K., Maki-Yonekura, S. & Namba, K. Complete atomic model of the bacterial flagellar filament by electron cryomicroscopy. *Nature* **424**, 643-650, doi:10.1038/nature01830 (2003).
5. Parkinson, J. S. Complementation analysis and deletion mapping of *Escherichia coli* mutants defective in chemotaxis. *Journal of bacteriology* **135**, 45-53 (1978).
6. Datsenko, K. A. & Wanner, B. L. One-step inactivation of chromosomal genes in *Escherichia coli* K-12 using PCR products. *Proceedings of the National Academy of Sciences of the United States of America* **97**, 6640-6645, doi:10.1073/pnas.120163297 (2000).
7. Sowa, Y. *et al.* Direct observation of steps in rotation of the bacterial flagellar motor. *Nature* **437**, 916-919, doi:10.1038/nature04003 (2005).
